# Paternal preconception modifiable risk factors for adverse pregnancy and offspring outcomes: a review of contemporary evidence from observational studies

**DOI:** 10.1186/s12889-023-15335-1

**Published:** 2023-03-16

**Authors:** Tristan Carter, Danielle Schoenaker, Jon Adams, Amie Steel

**Affiliations:** 1 School of Public Health, Faculty of Health, University of Technology Sydney, Sydney, 2006 Australia; 2 School of Primary Care, Population Sciences and Medical Education, Faculty of Medicine, University of Southampton, Southampton, UK; 3 NIHR Southampton Biomedical Research Centre, University of Southampton and University Hospital Southampton NHS Foundation Trust, Southampton, UK

**Keywords:** Paternal, Preconception, Modifiable, Risk factor, Pregnancy outcomes, Offspring outcomes

## Abstract

**Background:**

The preconception period represents transgenerational opportunities to optimize modifiable risk factors associated with both short and long-term adverse health outcomes for women, men, and children. As such, preconception care is recommended to couples during this time to enable them to optimise their health in preparation for pregnancy. Historically, preconception research predominately focuses on maternal modifiable risks and health behaviours associated with pregnancy and offspring outcomes; limited attention has been given to inform paternal preconception health risks and outcomes. This systematic review aims to advance paternal preconception research by synthesising the current evidence on modifiable paternal preconception health behaviours and risk factors to identify associations with pregnancy and/or offspring outcomes.

**Methods:**

Medline, Embase, Maternity and Infant care, CINAHL, PsycINFO, Scopus, and ISI Proceedings were searched on the 5^th^ of January 2023, a date limit was set [2012–2023] in each database. A Google Scholar search was also conducted identifying all other relevant papers. Studies were included if they were observational, reporting associations of modifiable risk factors in the preconception period among males (e.g., identified as reproductive partners of pregnant women and/or fathers of offspring for which outcomes were reported) with adverse pregnancy and offspring outcomes. Study quality was assessed using the Newcastle–Ottawa Scale. Exposure and outcome heterogeneity precluded meta-analysis, and results were summarised in tables.

**Results:**

This review identified 56 cohort and nine case control studies. Studies reported on a range of risk factors and/or health behaviours including paternal body composition (*n* = 25), alcohol intake (*n* = 6), cannabis use (*n* = 5), physical activity (*n* = 2), smoking (*n* = 20), stress (*n* = 3) and nutrition (*n* = 13). Outcomes included fecundability, IVF/ISCI live birth, offspring weight, body composition/BMI, asthma, lung function, leukemia, preterm birth, and behavioural issues. Despite the limited number of studies and substantial heterogeneity in reporting, results of studies assessed as good quality showed that paternal smoking may increase the risk of birth defects and higher paternal BMI was associated with higher offspring birthweight.

**Conclusion:**

The current evidence demonstrates a role of paternal preconception health in influencing outcomes related to pregnancy success and offspring health. The evidence is however limited and heterogenous, and further high-quality research is needed to inform clinical preconception care guidelines to support men and couples to prepare for a health pregnancy and child.

**Supplementary Information:**

The online version contains supplementary material available at 10.1186/s12889-023-15335-1.

## Plain English Summary

The time prior to conception, preconception, is widely acknowledged as an integral period whereby a woman’s health, lifestyle, and diet influence the outcomes of future pregnancy and the health of future offspring. Similarly, the influence of a man’s health, lifestyle, and diet during preconception on pregnancy and offspring outcomes must be considered. However, the male reproductive partner’s role during preconception has attracted much less researcher attention when compared to maternal exposures and outcomes and may be undervalued.

Therefore, this review explores the modifiable risk factors of males in the preconception period and how these risks influence adverse pregnancy and/or offspring outcomes. A total of 65 papers are included for review which examined risks associated with factors such as alcohol use, physical activity, stress, and nutrition. Overall, the papers identified some consistent results: paternal smoking increased risk of adverse offspring outcomes, while increased paternal body mass index was associated with higher offspring birthweight. Nevertheless, this review concludes that paternal preconception modifiable risk factors remain largely underexplored. Evidently, more high-quality research must be conducted to better understand the health, lifestyle, and diets of males in the preconception period and how various paternal modifiable risks can influence their partner’s pregnancy and the health and developmental outcomes of their offspring.

## Introduction

Preconception care is defined as the provision of health interventions (behavioural, social, and/or biomedical) to women and couples prior to conception [1]. It addresses the transgenerational opportunity of enabling and optimizing health while limiting risk factors associated with both short- and long-term adverse health outcomes for women, men, and their children. There is global consensus on the key aspects of preconception care [2], yet a consistent definition and clear attributes of the preconception population remain elusive [3]. Preconception research predominately focuses on maternal modifiable risks or health behaviours associated with offspring outcomes [4] as demonstrated by a scoping review of preconception health behaviours which found only 11% of all studies included paternal modifiable risks or health behaviours [5]. Nonetheless, the research community recognizes the father or male partner’s contribution to child health and development before birth [6, 7] and the need to balance our gaze on men in preconception care [8]. This is further supported by the increasing number and diversity of publications about paternal preconception health [9] and formulation of the Paternal Origins of Health and Disease (POHaD) model [10]. As such, the preconception population may include all reproductively aged individuals in a period from their birth to the conception of their (or their partner’s) pregnancy. The care provided during this period must respond to a clear set of identified risk factors and exposures as relevant to each individual.

Indeed, when planning parenthood, males find themselves within a contentious grey zone; concurrently involved while also considered an outsider [11]. A recent survey in the UK found that men are interested in engaging in positive preconception health behaviours [7]. Of the over 500 men surveyed, 19% had visited a primary health provider for preconception health advice, and those who had received advice were more likely to adopt positive health behaviours before their partner’s pregnancy. On the other hand, general practitioners (GPs) report low confidence in their knowledge about paternal preconception health care and modifiable factors affecting male fertility [12, 13]. They describe feeling apprehensive or even sensitive to the subject matter and/or challenged by navigating the stereotypical masculine predispositions toward fertility and preconception care [14]. In general, preconception risks are not raised by GPs with male patients unless subfertility is involved and preconception discussions are often encumbered by numerous impediments including the limited time, financial constraints, and knowledge of GPs, plus in some cases, a lack of GP motivation and perceived need for health care [12]. A systematic review of preconception care guidelines found that six of the 11 guidelines included provided preconception care guidance for men [15]. Only one guideline, a position paper from the American Academy of Family Physicians, contained a dedicated section outlining recommendations on preconception interventions for men [16]. Evidently, there is an unmet need for health professionals, and men, to readily access current relevant information regarding paternal preconception health exposures and outcomes, informing clinical practice and directing health decisions.

Evidence supporting paternal preconception care considers males contribution to child health and development before conception via direct (genetic and epigenetic contributions – health and lifestyle behaviours, exposure to environmental toxins, life stressors, and neuroendocrinology) and indirect pathways (the couple’s relationship, and the influence of men on their partner’s health and health behaviours) [17]. Yet, there is a stark contrast between the magnitude of research investigating maternal preconception health risks—including body composition, lifestyle behaviours, and diet/nutrition – and the relative scarcity of research attention directed towards understanding paternal health exposures and outcomes. In direct response, this systematic review aims to advance paternal preconception research by synthesising the current evidence on associations of modifiable paternal preconception health behaviours and risk factors with pregnancy and/or offspring outcomes.

## Methods

This review was prospectively registered in PROSPERO (Registration Number: CRD42021209994), and reported in line with PRISMA 2020 guidance [18] and the AMSTAR 2 critical appraisal tool [19].

### Search strategy

A search was conducted on January 5^th^ 2023, (See Supplementary File 1 Search strategy), through the following databases: 1) Medline (OVID) 2) Embase (OVID), 3) Maternity and Infant care [MIDIRS] (OVID) 4) CINAHL (EBSCO), 5) PsycINFO (EBSCO), 6) Scopus, & 7) ISI Proceedings. For each database, a date limit of 2012–2023 was set. When available, subject headings identified from the controlled vocabulary of each database were also included in the search. On January 11^th^ 2023, a Google Scholar search was conducted for the search term ‘Paternal preconception’, applying the filter to limit articles published since 2022 and searching through to page seven, identifying any other recently published relevant papers. Google Scholar was also used to identify relevant studies citing each included paper. Reference lists of each included paper were then checked for additional relevant studies.

### Selection criteria

Papers were included if they were original contemporary observational research (cross-sectional, cohort or case–control study designs) involving males in the preconception period, examining an association or correlation of a modifiable risk factor or health behaviour to pregnancy and/or offspring health and developmental outcomes. The male participants must identify as being the partner of the pregnant women and/or the biological father of the child for which pregnancy and offspring outcomes were reported (Table 1 – PICO).

**Table 1:** PICO (Population, Intervention, Comparison, Outcome) inclusion criteria

Population	Intervention/Exposure	Comparison	Outcome
Males who identified as being the partner of the pregnant women and/or the biological father of the child for which outcomes were reported	Exposure to modifiable risk factor(s) in the preconception period	No exposure to modifiable risk factor(s) in the preconception period (or comparison group as defined by individual studies)	Adverse pregnancy and offspring outcomes

Observational study designs are generally utilized to identify correlations and establish findings at the population level hence are solely considered in this review.

Papers were excluded if they were: reviews, did not report new empirical findings from original studies (i.e. commentaries, opinion-pieces and editorials), not studying humans, not examining male parent exposures, did not differentiate between maternal and paternal preconception exposures, or if the exposure examined specific illness populations. Papers were also excluded when the exposure was not assessed or retrospectively recalled during the preconception period, the outcome was not related to pregnancy or offspring health or development, or the risk factor or health behaviour was not modifiable. Google Translate was used to decipher any studies located in languages other than English.

### Data extraction

Papers were imported into Covidence systematic review software [20], and duplicates removed by automation. Titles and abstracts were screened by TC, AS and DS. Full-text articles were obtained for relevant studies and reviewed based on inclusion criteria by TC who then extracted data from each included paper. AS or DS randomly reviewed the extracted data of ten included studies for accuracy and completeness. Any conflicts were resolved by consensus.

Data extracted from each paper included: the authors and year, study design and duration, location, the preconception population, total number of participants, the paternal exposures (and exposure measures), paternal outcomes (and outcome measures), any covariates considered and the main results from each association reported.

### Quality assessment

The quality of each paper was assessed by TC using the Newcastle–Ottawa Scale (NOS). The NOS comprises three domains 1) selection of participants, 2) comparability of study groups, and 3) outcome of interest (cohort studies) or ascertainment of exposure (case–control studies), assigning stars in each domain to a maximum of nine stars [21]. Papers were then categorized as good quality (7–9 stars), fair quality (4–6 stars) or poor quality (0–3 stars) using groupings employed in previous research [22].

A meta-analysis was considered, but not possible due to exposure and outcome heterogeneity.

## Results

**Fig. 1:**
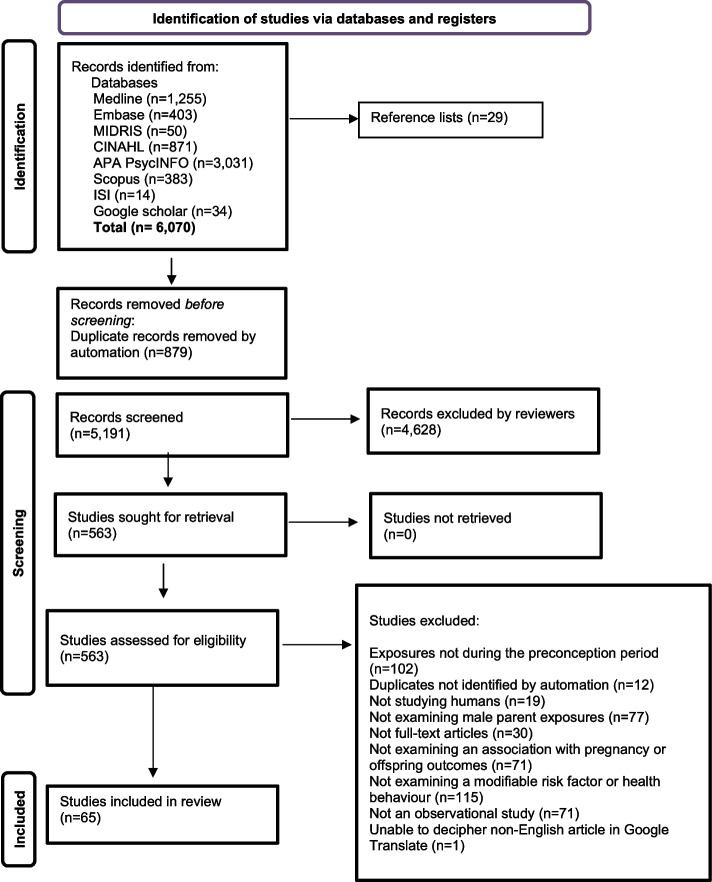
PRISMA Flowchart

A total of 65 papers were included in this review (Fig. 1 – PRISMA Flowchart) [18], comprising cohort (*n* = 56) and case control studies (*n* = 9) (Table 2 – Summary Table) & (Table 3 – Summary Table Findings). The majority of papers were conducted in the USA (*n* = 18), Europe and the UK (*n* = 19), and China (*n* = 17), several papers were from Australia [23–29] or included an Australian health centre [30–34]. Approximately half of all papers (*n* = 29) included a sample size between 370 and 2,900, while others included > 20,000 (*n* = 11) or ≤ 200 participants (*n* = 13).

**Table 2:** Summary table

**First Author & Year**	**Location**	**Design & Duration**	**Sample**	**Exposure Measure**	**Paternal Exposure**	**Confounders**	**Outcome Measure**	**Outcome**	**Quality**^±^
**Body Composition**									
Bowatte et al. 2022 [25]	Australia	Cohort [Prospective] Tasmanian Longitudinal Health Study (TAHS) 1968—2021	Mothers & **Fathers (*N*** **= 836)** of offspring (*n* = 1,938)	Paternal height and weight obtained from school medical records	*BMI –* BMI trajectory from early childhood (4–6 years) to late childhood (9–10 years) and adolescence (14–15 years)	1)Maternal report of asthma at 14 years 2) Paternal report of asthma at 14 years 3) Grandfather or Grandmother ever asthma 4) Smoking status of Grandfather or Grandmother during paternal childhood 5) Grandfather’s occupation	1) ‘Ever’ Allergic asthma 2) Asthma onset before 10 years old 3) Asthma onset after 10 years old	Offspring asthma	5
Broadney et al. 2017 [35]	USA	Cohort [Retrospective] Upstate KIDS Study (Population-based) 2008–2010	Mothers & **Fathers (*N* = 2,974)** of infants (*n* = 3,555)	Maternal report of paternal weight & height on baseline questionnaire at 4 months postpartum	*BMI*—"Pre-pregnancy" body mass index [BMI] (Weight in kilograms over height in meters squared)	1) Maternal age, 2) Race/ethnicity, 3) Education, 4) Private insurance, 5) Maternal smoking during pregnancy, 6) Alcohol use during pregnancy, 7) Parity, 8) Infant plurality, 9) Maternal pre-pregnancy BMI	Inflammatory biomarker [CRP] and Ig levels	Inflammation & immune response of neonates	6
Casas et al. 2017 [36]	Spain	Cohort [Prospective] INfancia y Medio Ambiente- Environment and Childhood [INMA] (Population-based) 2003–2008	Pregnant couples & their expectant children **(*N*** **= 1,827)**	Maternal report of paternal weight & height at first prenatal visit approximately 14 weeks of gestation	*BMI*—"Pre-pregnancy" body mass index [BMI] (Weight in kilograms over height in meters squared)	1) Parental age, 2) Sex of the child, 3) Parental education, 4) Parental social class, 5) Parity, 6) Maternal IQ, 7) Maternal employment status during pregnancy and at 5 years, 8) Breastfeeding duration, 9) Daycare attendance, 10) Child physical activity, 11) Maternal BMI	**1)** McCarthy Scales of Children's Abilities (MSCA) [contexualized to Spanish], & **2)** The attention deficit hyoperactivity disorder [ADHD] Criteria of Diagnostic and Statistical Manual of Mental Health Disorders—4th Edition (ADHD-DSM-IV)	Neuropsychological development of preschool children around 5 years old	9
Chen et al. 2021 [37]	China	Cohort [Retrospective] Women’s Hospital, School of Medicine, Zhejiang University (Hospital-based) 2013—2016	Subfertile couples (**Males [*N* = 2,318])** undergoing IVF/ICSI fresh embryo transfer cycles resulting in singletons (*n* = 1,366) and twins (*n* = 952)	Third Party—Measurement of paternal weight and height by trained nurse	*BMI—*"Pre-pregnancy" body mass index [BMI] (Weight in kilograms over height in meters squared)	1)Parental age, 2) type of infertility, 3) duration of infertility, 4) ovulatory dysfunction, 5) endometriosis, 6) maternal prepregnancy BMI	International classification of Diseases, 10^th^ Revision (ICD-10) into 9 subcategories	Birth defect	5
Fang et al. 2020 [38]	China	Cohort [Retrospective] National Free Preconception Health Examination Project (NFPHEP) (Population-based) 2012–2016	Couples planning to conceive **[Males [*N* = 50,927])**	Third Party—Measurement of paternal weight and height by physician	*BMI—*"Pre-pregnancy" body mass index [BMI] (Weight in kilograms over height in meters squared)	1)Age, 2) type of household, 3) education, 4) smoking, 5) alcohol consumption, 6) psychosocial pressure and ready for pregnancy 7) cycle regularity, 8) age of menarche, 9) gravidity, 10) spontaneous abortion, 11) induced abortion	Time to pregnancy (TTP) = interval between the date of enrolment and last menstrual period (LMP)	Fecundability	5
Fleten et al. 2012 [39]	Norway	Cohort [Prospective] Norwegian Mother and Child cohort study (MoBa) (Population-based) 1999–2009	Pregnant couples & their expectant children **(*N* = 29,216)**	Paternal self-report of weight and height (20%) OR maternal report of paternal weight and height (80%) at approximately 17 weeks of gestation	*BMI—*"Pre-pregnancy" body mass index [BMI] (Weight in kilograms over height in meters squared)	1) Parental educational level (years), 2) Paternal and maternal prenatal smoking, 3) Maternal coffee consumption during pregnancy, 4) Parental BMI	Body mass index (BMI) at 3 years old	Offspring adiposity	6
Guo et al. 2022 [40]	China	Cohort [Retrospective] National Free Pre-conception Check-up Projects (NFPCP) 2013–2017	Nulliparous couples attempting pregnancy **(Males [*N* = 4,719,813])**	Third Party—Physician measurement of paternal weight and height	*BMI—*"Pre-pregnancy" body mass index [BMI] (Weight in kilograms over height in meters squared) during participation in the NFPCP	1)Maternal and paternal age at last menstrual period, 2) Maternal and paternal height 3) Education level, 4) Parity, 5) Ethnicity, 6) Area of residence 7) Maternal Diabetes, 8) Maternal Hypertension, 9) Smoking 10) Alcohol use 11) Passive smoking 12) History of adverse pregnancy incl preterm birth, stillbirth, or spontaneous abortion in previous pregnancies	**1)** Large-for-gestational- age (LGA) = birthweight above 90th percentile according to birthweight centiles for a Chinese population, & **2)** Small-for-gestational-age (SGA) = birthweight below the tenth percentile on birthweight centiles for a Chinese population	Offspring birthweight	6
Hoek et al. 2022 [41]	The Netherlands	Cohort [Prospective] Rotterdam Periconception Cohort (PREDICT Study) (Hospital-based) 2017–2019	Subfertile couples (**Males [N = 221])** undergoing IVF/ICSI with cultured embryos (*n* = 757)	Third party—Anthropometric assessment completed by a trained nurse at baseline	*BMI*—"Preconceptional" body mass index [BMI] (Weight in kilograms over height in meters squared)	1) Total motile sperm count [TMSC], 2) Age, 3) Ethnicity, 4) Smoking, 5) Alcohol use, 6) Education	**1)** Fertilization rate, **2)** TMSC **3)** Embryo developmental morphokinetics, **4)** Embryo quality assessed by a time-lapse prediction algorithm (KIDScore), & **5)** Live birth rate	IVF/ICSI induced live birth	8
Johannessen et al. 2020 [33]	**Northern Europe** Denmark Norway Sweden Iceland Estonia & Spain Australia	Cohort [Prospective] The Respiratory Health in Northern Europe, Spain and Australia multigeneration study (RHINESSA) (Population-based) 2013–2016	Mothers & **Fathers (*N* = 2044),** of adult offspring (*n* = 2,822)	Paternal self-report based upon validated figural drawing scale of 9 sex-specific silhouettes	*BMI—*“Overweight status” To identify subjects at risk for overweight body size (BMI, 25–30 kg/m^2^) at 8 years old, at puberty, and at age 30 years before offspring conception	1)Paternal asthma status, 2) Education level 3) Maternal overweight status 4) Maternal asthma status 5) Offspring sex 6) Offspring age	Parent report in the RHINESSA questionnaire	Adult offspring asthma with or without nasal allergies	6
Lonnebotn et al. 2022 [34]	**Northern Europe** Denmark Norway Sweden Iceland Estonia & Spain Australia	Cohort [Prospective] The Respiratory Health in Northern Europe, Spain and Australia multigeneration study (RHINESSA) (Population-based) 2013–2016	Mothers & **Fathers (*N* = 308)** of adult offspring (*n* = 420)	Paternal self-report based upon validated figural drawing scale of 9 sex-specific silhouettes	*BMI—*“Overweight status” To identify subjects at risk for overweight body size (BMI, 25–30 kg/m^2^) at 8 years old and at puberty	1)Maternal education 2) Paternal education 3) Offspring age 4) Smoking history	Pre/post bronchodilator forced expiratory volume in one second (FEV1) & forced vital capacity (FVC)	Adult offspring lung function	7
Moss et al. 2015^a^ [42]	USA	Longitudinal cohort [Prospective] National Longitudinal Study of Adolescent Health (Add Health) 1994–2008	Adolescents (grades 7 -12) followed into adulthood becoming Mothers & **Fathers** of infants** (*N* = 372**)	Third party—Anthropometric assessment completed by a trained professional at baseline	*BMI—*"Preconception" body mass index [BMI] (Weight in kilograms over height in meters squared)	1) Parents age at birth, 2) Race/ethnicity, 3) Immigrant status, 4) Education level, 5) Socioeconomic status, 6) Infant sex, 7) Initiation of prenatal care, 8) Parity, 9) Time between wave III interview and conception, 10) Relationship type at wave III	Respondent self-report on Wave IV questionnaire	Gestational age & offspring birthweight	7
Mutsaerts et al. 2014^a^ [43]	The Netherlands	Cohort [Prospective] Groningen Expert Center for Kids with Obesity [GECKO] Drenthe cohort (Population-based) 2006–2007	Pregnant couples & their expectant children** (*N* = 2,264**)	Paternal self-report of weight and height on baseline questionnaire during third trimester or within 6 months postpartum	*BMI—*"Prepregnancy" Body mass index [BMI] at conception	Nil	Questionnaire, shortly after birth, completed by midwife or gynaecologist	Spontaneous preterm birth & Small for gestational age (SGA)	3
Noor et al. 2019 [44]	USA	Longitudinal cohort [Prospective] Project Viva birth cohort study of mothers and children 1999–2019	Pregnant couples & their expectant children** (*N* = 429**)	Maternal report of paternal weight & height at first prenatal visit approximately 10 weeks gestation	*BMI*—"Periconception" body mass index [BMI] (Weight in kilograms over height in meters squared)	1) Maternal prepregnancy BMI, 2) Maternal Age, 3) Gestational weight gain, 4) Household income, 5) Maternal education, 6) Maternal smoking, 7) Maternal alcohol use, 8) Marital status, 9) Infant's sex, 10) Race/ethnicity, 11) Gestational age at delivery, 12) Mode of delivery, 13) Birth weight, 14) Batch effects, 15) Estimated nucleated cell types from cord blood 16) WBC's	Blood samples collected at birth, age 3 years & 7 years	Genome-wide DNA methylation patterns and birthweight in offspring	7
Pomeroy et al. 2015 [23]	Australia	Cohort [Prospective] Mater-University of Queensland Study of Pregnancy (MUSP) 1982–1983	Mothers and **Fathers** of infants **(*N*** **= 1,041)**	Maternal report of paternal weight and height at first prenatal visit approximately 18 weeks of gestation	*BMI—*"Pre-pregnancy" height & body mass index [BMI] (Weight in kilograms over height in meters squared)	1) Parity, 2) Maternal education, 3) Maternal smoking in the last trimester, 4) Maternal age at birth	**1)** Birthweight, **2)** Neck-rump length 3) Head circumference, **3)** Absolute and proportional limb segment and trunk lengths & **4)** Subcutaneous fat	Neonatal body measurements	6
Retnakaran et al. 2021 [45]	China	Cohort [Prospective] Liuyang Preconception cohort 2009 -	Newly married couples attempting pregnancy and their expectant children **(*N* = 1,292)**	Third party—Anthropometric assessment completed by trained staff at baseline	*BMI—*"Pregravid" body mass index [BMI] (Weight in kilograms over height in meters squared)	1) Age, 2) Years of education, 3) Smoking status, 4) BMI, 5) Household income 6) Length of gestation, 7) Total gestational weight gain, 8) Gestational diabetes, 9) Preeclampsia, & 10) Infant sex	**1)** Large-for-gestational- age (LGA) = birthweight above 90th percentile according to birthweight centiles for a Chinese population, & **2)** Small-for-gestational-age (SGA) = birthweight below the tenth percentile on birthweight centiles for a Chinese population	Offspring birthweight	8
Robinson et al. 2020 [46]	USA	Cohort [Prospective] Upstate KIDS study (Population-based) 2008–2010	Mothers and **Fathers** of children** (*N*** **= 1,915)**	Maternal report of paternal weight & height on baseline questionnaire at 4 months postpartum	*BMI—*"Pre-pregnancy" body mass index [BMI] (Weight in kilograms over height in meters squared)	1) Maternal & paternal age, 2) Insurance status, 3) Child sex, 4) Maternal race/ethnicity, 5) Education, 6) Marital status, 7) History of polycystic ovary syndrome (PCOS) and/or diagnosis, 8) Smoking, 9) Alcohol intake, 10) Maternal & paternal history of affective disorders, 11) BMI, 12) Maternal prepregnancy BMI	**1)** Positive history of attention deficit hyperactivity disorder (ADHD) or anxiety disorder **2)** Positive screening for ADHD and the inattentive or hyperactive/impulse sub scales OR report of clinical ADHD diagnosis **3)** Parental report of child borderline behavioural problems at 7 or 8 years of age	Offspring behavioural problems and psychiatric symptoms at 7–8 years	7
Sun et al. 2022 [47]	China	Cohort [Prospective] Hunan Maternal and Child Health Hospital (Hospital-based) 2013–2019	Couples receiving antenatal care **(Males [*N* = 34,104)**	Third party – Paternal height and weight measured at 14–16 weeks gestation	*BMI—*"Pre-pregnancy" body mass index [BMI] (Weight in kilograms over height in meters squared)	1)Paternal age, 2) maternal age, 3) maternal BMI, 4) residence location, 5) education level, 6) nationality, 7) history of smoking, 8) history of drinking, 9) history of betel nut consumption, 10) history of drug use, 11) history of preterm birth, 12) per capita monthly household income	Delivery before 37 weeks gestation & Birth weight < 2,500 g	Preterm birth & Low birth weight	7
Sundaram et al. 2017 [48]	USA	Cohort [Prospective] Longitudinal Investigation of Fertility and the Environment [LIFE]) 2005–2009	Couples attempting pregnancy **(Males [*N* = 501])**	Third party—Anthropometric assessment completed by a trained nurse at baseline	*BMI—*Body mass index [BMI] (Weight in kilograms over height in meters squared) and waist/hip measurements	1) Female partner's age, 2) Difference between the male and female age, 3) Both partner's smoking status, 4) Both partner's number of days of vigorous physical activity per week, 5) Both partner's free cholesterol level 6) Both partner's race 7) Both partner's education 8) Average acts of intercourse per menstrual cycle 9) Menstrual cycle regularity	Time to pregnancy (TTP) in menstrual cycles	Pregnancy	8
Umul et al. 2015 [49]	Turkey	Cohort [Retrospective]	Couples **(Males [*N* = 155])** undergoing intracytoplasmic sperm injection (ICSI) cycles (n = 177)	Third party—Anthropometric measurements	*BMI—B*ody mass index [BMI] (Weight in kilograms over height in meters squared) during fertility treatment	Nil	**1)** Fertilization rate, **2)** Implantation rate, **3)** Clinical pregnancy rate, & **4)** Live birth rate	ICSI induced live birth	2
Wei et al. 2022 [50]	China	Cohort [Prospective] Hunan Provincial Maternal and Children Health Care Hospital (Hospital-based) 2013–2019	Pregnant couples **(Males [*N* = 40,650])**	Paternal self-report of weight and height on baseline antenatal questionnaire between 8- and 14-weeks’ gestation	*BMI—*"Pre-pregnancy" body mass index [BMI] (Weight in kilograms over height in meters squared)	1)Maternal and paternal age 2) ethnicity, 3) educational level, 4) parity, 5) family income per month, 6) active smoking before pregnancy, 7) passive smoking before pregnancy, 8) alcohol consumption before pregnancy 9) folic acid consumption before or during pregnancy, 10) history of adverse pregnancy outcomes, 11) history of pregnancy complications, 12) gestational weight gain recommendation range, 13) pregnancy complications in this pregnancy, 14) smoking status before pregnancy, 15) alcohol consumption before pregnancy	Low birth weight = < 2,500 g Very low birthweight = < 1,500 g Extremely low birthweight < 1,000 g	Offspring birthweight	6
Wei et al. 2021 [51]	China	Cohort [Retrospective] Guangxi Zhuang Birth Cohort (GZBC) (Hospital-based) 2015–2018	Parents with singleton birth **(Males [*N* = 1,082])**	Paternal self-report of weight and height at first antenatal interview	*BMI—*"Pre-pregnancy" body mass index [BMI] (Weight in kilograms over height in meters squared)	1)Parental age at delivery, 2) offspring sex, 3) gestational age, 4) offspring birth weight), 5) maternal residential place, 6) gravidity, 7) parity, 8) drinking before pregnancy, 9) maternal passive smoking during pregnancy, 10) pregnancy comorbidities or complications, 11) caesarean section	Real-time polymerase chain reaction (qPCR)	Newborn telomere length (TL)	6
Xu et al. 2021 [52]	China	Cohort [Prospective] Shanghai Jiao Tong University 2015	Pregnant couples and their expectant children **(*N* = 1,810)**	Paternal self-report of weight and height at first prenatal visit approximately 16 weeks of gestation	*BMI—*"Preconception" body mass index [BMI] (Weight in kilograms over height in meters squared) during fertility treatment	1) Delivery gestational week, 2) Maternal age, 3) Gestational weight gain (GWG), 4) Education, 5) Parity, 6) Family history of metabolic diseases, 7) Haemoglobin, 8) Systolic blood pressure, 9) Diastolic blood pressure, 10) Dyslipidemia, 11) Fasting plasma glucose at the first prenatal check-up 12) Offspring sex 13) Preconception BMI	Assessed within 1 h of birth using digital scales	Offspring birthweight	7
Yang et al. 2015 [53]	China	Case–control [Retrospective] (Population-based) 2011–2013	Mothers & **Fathers** of cases **(*N* = 870)** and controls **(*N* = 5,471)**	Paternal self-report of weight and height at postpartum baseline interview	*BMI—*"Pre-pregnancy" body mass index [BMI] (Weight in kilograms over height in meters squared)	1) Infant's gender, 2) Gestational age, 3) Parental age, 4) Family income, 5) Parental education level, 6) Gravidity, 7) Parity, 8) Paternal smoking status during pregnancy, 9) Parental prepregnancy weight,10) Parental height, 11) Parental BMI, 12) Maternal alcohol consumption during pregnancy, 13) Maternal weight gain during pregnancy, 14) Maternal BMI gain during pregnancy	Live macrosomic birth (> 4,000 g)	Macrosomia	6
Zalbahar et al. 2017 [24]	Australia	Cohort [Prospective] Mater-University of Queensland Study of Pregnancy (MUSP) 1981–1983	Mothers and **Fathers** of infants** (*N* = 1,494)**	Maternal report of paternal weight and height at first prenatal visit at approximately 18 weeks of gestation	*BMI—*"Pre-pregnancy" weight and body mass index [BMI] (Weight in kilograms over height in meters squared)	1) Parental education, 2) Family annual income, 3) Maternal gestational weight gained, 4) Maternal smoking habit, 5) Offspring birth weight, 6) Offspring gender, 7) Gestational age, 8) Breastfeeding duration, 9) Offspring's lifestyle at 14 years, 10) Maternal or paternal BMI, 11) Maternal age at birth, 12) Offspring birth weight, 13) Offspring gender	Physical assessment using measuring tape and digital scales at 5, 14 and 21 year follow-ups	Offspring weight & BMI changes from childhood (5 years) into adulthood (21 years)	5
Zhang et al. 2020 [54]	China	Cohort [Retrospective] National Free Pre-conception Check-up Projects (NFPCP) 2015–2017	Nulliparous couples attempting pregnancy **(Males [*N*** **= 2,301,782])**	Third Party—Physician measurement of paternal weight and height	*BMI—*"Pre-pregnancy" body mass index [BMI] (Weight in kilograms over height in meters squared) during participation in the NFPCP	1) Age, 2) Ethnic background, 3) Educational level, 4) Occupation, 5) Household registration and region, 6) Alcohol intake, 7) Tobacco exposure, 8) Hypertension, 9) HBsAg positive status based on male individual model A	Time to pregnancy (TTP) = [Date of the last menstruation (pregnant couples) or Date of the most recent follow-up (nonpregnant couples) - Date of baseline questionnaire completion)]/Average menstrual cycle length] + 1	Pregnancy	9
**Alcohol**									
Luan et al. 2022 [55]	China	Cohort [Prospective] Shanghai-Minhang Birth Cohort Study 2012 -	Mothers and Fathers of infants **(*N* = 796)**	Maternal report of paternal preconception alcohol consumption at 12–16 weeks gestation	*Alcohol –* 3 months before conception	1)Paternal age 2) Paternal BMI 3) Paternal education 4) Paternal smoking 5) Maternal age 6) Parity 7) Maternal depressive symptoms during pregnancy 8) Maternal preconception folic acid supplements, 9) Multivitamin supplements during pregnancy 10) Gestational weeks 11) Sex	Child Behaviour Checklist (CBCL) at offspring ages 2, 4, & 6 years old	Offspring behavioural problems	7
Milne et al. 2013 [26]	Australia	Case–control [Retrospective] Aus-ALL 2003–2006 Aus-CBT 2005–2010	Mothers and **Fathers** of children with ALL (Cases [*n* = 281] Controls [*n* = 672) & CBTs (Cases [*n* = 221]) and Controls [*n* = 717]	Paternal self-report on baseline questionnaire	*Alcohol –*Any alcohol 12 months before pregnancy	1)Year of birth group 2) Maternal age, 3) Ethnicity 4) Household income 5) Birth order 6) Maternal smoking 7) Child’s age 8) Child’s sex 9) State of residence 10) Paternal smoking 11) Paternal age group 12) Household income	Diagnosis from one of 10 paediatric oncology centres in Australia	Childhood acute lymphoblastic leukemia (ALL) & Childhood brain tumours (CBTs)	6
Moss et al. 2015^a^ [42]	USA	Longitudinal cohort [Prospective] National Longitudinal Study of Adolescent Health (Add Health) 1994–2008	Adolescents (grades 7 -12) followed into adulthood becoming Mothers & **Fathers** of infants** (*N* = 372**)	Paternal self-report of health behaviours at wave III interview	*Alcohol—*preconception intake greater than once a month	1) Parents age at birth, 2) Race/ethnicity, 3) Immigrant status, 4) Education level, 5) Socioeconomic status, 6) Infant sex, 7) Initiation of prenatal care, 8) Parity, 9) Time between wave III interview and conception, 10) Relationship type at wave III	Respondent self-report on Wave IV questionnaire	Gestational age & offspring birthweight	7
Mutsaerts et al. 2014^a^ [43]	The Netherlands	Cohort [Prospective] Groningen Expert Center for Kids with Obesity [GECKO] Drenthe cohort (Population-based) 2006–2007	Pregnant couples & their expectant children** (*N* = 2,264**)	Paternal self-report on baseline questionnaire during third trimester or within 6 months following delivery	Alcohol intake (units/week) 6 months prior to conception and up to delivery	Nil	Questionnaire, shortly after birth, completed by midwife or gynaecologist	Spontaneous preterm birth & Small for gestational age (SGA)	3
Xia et al. 2018 [56]	China	Cohort [Prospective] Shanghai-Minhang Birth Cohort Study 2012	Mothers and F**athers** of infants **(*N* = 980)**	Paternal self-report at baseline interview between 12 to 16 weeks of gestation	*Alcohol—*intake at least once a week 3 months before conception	1) Parental age, 2) Parental BMI before conception, 3) Gestational age, 4) Gravidity, 5) Birth weight of offspring, 6) Paternal education, 7) Maternal passive smoking before conception (yes/no), 8) Paternal smoking (yes/no), 9) Days between birth and 12-month measurement	**Males—**AGD-AP (centre of anus to penis) AGD-AS (centre of anus to scrotum) **Females—**AGD-AC (centre of anus to clitrous) AGD-AF (centre of anus to fourchette)	Offspring anogenital distance (AGD)	8
Zuccolo et al. 2016 [57]	Norway	Cohort [Prospective] The Norwegian Mother and Child Cohort Study (MoBa) (Population based) 1999–2009	Mothers & F**athers** of children **(*N* = 68,244)**	Paternal self-report on baseline questionnaire at approximately 17 weeks of gestation	*Alcohol—*intake in the 6 months prior to pregnancy and up to week 18 of gestation	1) Year of birth, 2) Folic acid use around conception, 3) Whether the pregnancy was planned, 4) Maternal diabetes, 5) Parity, 6) Ethnicity, 7) Financial strain, 8) Parental age, 9) Height, 10) BMI, 11) Gross income, 12) Education, 13) Smoking/drug use in pregnancy, 14) Other parent's exposure	Sex-standardised head circumference (expressed as standard deviation [SD] scores), based on the distribution of all MoBa newborns by sex	Offspring head circumference	4
**Cannabis**									
Har-Gil et al. 2021 [58]	Canada	Cohort [Retrospective] (Clinic-based) 2016–2019	Female (*n* = 15) & **male (*N* = 53)** cannabis users & non-users** (*N* = 654)** undergoing IVF	Paternal self-report on baseline questionnaire	*Cannabis*—use prior to fertility treatment	Nil	1) Sperm volume 2) Sperm quality, 3) Fertilization rate 4) Implantation rate (IR) 5) Ongoing pregnancy rate (OPR)	IVF/ICSI induced live birth	2
Kasman et al. 2018 [59]	USA	Cross sectional cohort [Retrospective] National Survey of Family Growth (NSFG) (Population-based) 2002–2015	Female (*n* = 1,076) **& male (*N* = 758)** respondents of the National Survery of Family Growth (NSFG)	Paternal self-report at baseline interview	*Cannabis—*use over the previous 12 months	1) Age, 2) Marital status, 3) Previous children, 4) Partner age (for men), 5) Previous fertility evaluation/treatment, 6) Year of survey, 7) Income, 8) Race, 9) Education	Estimated time to pregnancy (TTP) using the current-duration appaorach	Pregnancy	6
Moss et al. 2015^a^ [42]	USA	Longitudinal cohort [Prospective] National Longitudinal Study of Adolescent Health (Add Health) 1994–2008	Adolescents (grades 7 -12) followed into adulthood becoming Mothers & **Fathers** of infants** (*N* = 372**)	Paternal self-report of health behaviours at wave III interview	*Cannabis*—use in the last 12 months	1) Parents age at birth, 2) Race/ethnicity, 3) Immigrant status, 4) Education level, 5) Socioeconomic status, 6) Infant sex, 7) Initiation of prenatal care, 8) Parity, 9) Time between wave III interview and conception, 10) Relationship type at wave III	Respondent self-report on Wave IV questionnaire	Gestational age & offspring birthweight	7
Nassan et al. 2019 [60]	USA	Cohort [Prospective] Environment and Reproductive Health Study [EARTH] 2005–2017	Subfertile couples (**Males [*N*** **= 200])** undergoing IVF cycles (*n* = 368)	Paternal self-report on baseline questionnaire	*Cannabis—*use ever	1) Age, 2) Race, 3) BMI, 4) Tobacco smoking, 5) Coffee and alcohol consumption, 6) Cocaine use	**1)** Implantation, **2)** Clinical pregnancy, **3)** Live birth per assisted reproductive technology (ART) cycle, & **4)** Pregnancy loss	IVF/ICSI induced live birth	7
Wise et al. 2018 [61]	USA	Cohort [Prospective] Preconception pregnancy planner cohort study online (PRESTO) 2013–2017	Couples attempting pregnancy **(Males *N* = 1,125)**	Paternal self-report on baseline questionnaire	*Cannabis—*use in the previous 2 months	1) Age, 2) Race/ethnicity, 3) Education, 4) Annual household income, 5) Cigarette smoking history, 6) Alcohol intake, 7) Caffeine intake, 8) Intercourse frequency, 9) Doing something to improve chances of conception, 10) PSS-10 score, 11) MDI score, 12) Sugar-sweetened soda intake, 13) Average sleep duration 14) Employment status	Time to pregnancy (TTP) = (Menstrual cycles of attempt at study entry) + [(Last menstrual period [LMP] date from the most recent followup questionnaire − date of baseline questionnaire completion)/usualmenstrual cycle length] + 1	Fecundability	6
**Physical activity**									
Moss et al. 2015^a^ [42]	USA	Longitudinal cohort [Prospective] National Longitudinal Study of Adolescent Health (Add Health) 1994–2008	Adolescents (grades 7 -12) followed into adulthood becoming Mothers & **Fathers** of infants** (*N* = 372**)	Paternal self-report of health behaviours at wave III interview	*Physical activity—*sessions in the last week	1) Parents age at birth, 2) Race/ethnicity, 3) Immigrant status, 4) Education level, 5) Socioeconomic status, 6) Infant sex, 7) Initiation of prenatal care, 8) Parity, 9) Time between wave III interview and conception, 10) Relationship type at wave III	Respondent self-report on Wave IV questionnaire	Gestational age & offspring birthweight	7
Mutsaerts et al. 2014^a^ [43]	The Netherlands	Cohort [Prospective] Groningen Expert Center for Kids with Obesity [GECKO] Drenthe cohort (Population-based) 2006–2007	Pregnant couples & their expectant children** (*N* = 2,264**)	Paternal self-report on baseline questionnaire during third trimester or within 6 months following delivery	*Physical activity*—moderate intensity for 30 min per day ≥ once a week 6 months prior to conception and up to delivery	Nil	Questionnaire, shortly after birth, completed by midwife or gynaecologist	Spontaneous preterm birth & Small for gestational age (SGA)	3
**Smoking**									
Accordini et al. 2021 [32]	**Northern Europe** Denmark Norway Sweden Iceland Estonia & Spain Australia	Cohort [Prospective] The Respiratory Health in Northern Europe, Spain and Australia multigeneration study (RHINESSA) (Population-based) 2013–2016	Mothers & **Fathers (*N* = 274),** investigated in the European Community Respiratory Health Survey (ECRHS), of adult offspring (*n* = 383)	Paternal self-report at baseline interview and ECRHS examinations	*Smoking –* Prepubertal smoking [smoking < 15 years old] & smoking ≥ 15 years old	1)Grand parents education level 2) Paternal age 3) Paternal education level 4) Paternal occupational class 5) Maternal smoking before or after offspring birth 6) Offspring age 7) Offspring sex 8) Offspring education level 9) Offspring smoking	Pre/post bronchodilator forced expiratory volume in one second (FEV1) & forced vital capacity (FVC)	Adult offspring lung function	8
Accordini et al. 2018 [31]	**Northern Europe** Denmark Norway Sweden Iceland Estonia & Spain Australia	Cohort [Prospective] European Community Respiratory Health Survey (ECRHS) (Population-based) 1998–2013	Mothers and **Fathers (*N* = 1,964)** of adult offspring (*n* = 4,192)	Paternal self-report at baseline interview and ECRHS examinations	*Smoking –*Prepubertal smoking [smoking < 15 years old] & smoking ≥ 15 years old	1)Grandmother smoking 2) Father’s ever asthma 3) Education level 4) Smoking initiation 5) Offspring gender 6) Age	Parent report in the ECRHS questionnaire	Adult offspring asthma with or without nasal allergies	7
Carslake et al. 2016 [62]	Norway	Combined cohort [Prospective] HUNT Study [Adult ≥ 20 years] (1984 – 2008)/ YoungHUNT Study [Child 13–19 years] (1995 – 2007)	Mothers and **Fathers (**[HUNT] of offspring [YoungHUNT] **(*N* = 221)**	Paternal self-report at baseline interview	*Smoking –*Prepubertal smoking [smoking < 11 years old]	1)Offspring birth order 2) Maternal education 3) Paternal employment 4) Maternal and Paternal smoking status at time of offspring conception 5) Offspring sex	Body Mass Index (BMI)	Offspring adiposity	6
Deng et al. 2013 [63]	China	Case–control [Retrospective] Gene-environmental interaction study on CHD occurrence (Hospital-based) 2010–2011	Pregnant couples & their expectant children as CHD cases **(*N* = 267)** & controls **(*N* = 386)**	Maternal report at baseline interview during pregnancy but after prenatal diagnosis of CHD	*Smoking*—"Periconceptional" being 3 months before conception through to the first trimester of pregnancy	1) Maternal residence, 2) Age, 3) Education, 4) Prepregnancy BMI, 5) Parental alcohol use during the 3 months before and 3 months after conception, 6) Folic acid intake during the 3 months before and 3 months after conception, 7) Family history of CHD, 8) Parity	Diagnosed via prenatal echocardiography	Congenital heart defects (CHD) in offspring	8
Frederiksen et al. 2020 [64]	Costa Rica	Case–control [Retrospective] Costa Rican Childhood Leukemia Study (CRCLS) (Population-based) 2001–2003	Mothers and Fathers (***N* = 198)** of offspring suffering leukemia (*N* = 292) [Cases] & cancer free age matched offspring (*N* = 578) [controls]	Paternal self-report at baseline interview	*Smoking –*Tobacco smoking 12 months before conception	1)Child sex 2) Birth year 3) Parental education 4) Paternal age 5) Maternal smoking	Diagnosis, between 1995–2000 in Costa Rica while aged < 15 years, of Acute Lymphoblastic Leukemia (ALL) (*N* = 252) or Acute Myeloid Leukemia (AML) (*N* = 40)	Childhood leukemia	7
Knudsen et al. 2020 [30]	**Northern Europe** Denmark Norway Sweden Iceland Estonia & Spain Australia	Cohort [Prospective] The Respiratory Health in Northern Europe, Spain and Australia multigeneration study (RHINESSA) (Population-based) 2013–2016	Mothers & **Fathers (*N* = 2,111)** of adult offspring (*n* = 2,939)	Paternal self-report at baseline interview and examinations	*Smoking –*Prepubertal smoking [smoking before 15 years old] & smoking ≥ 15 years old. Preconception smoking [≥ 2 years before offspring birth year]	1)Parental education 2) offspring sex	**1)** BMI [weight (kg)/height (m)^2^] **2)** Bioelectrical impedance analysis **3)** Fat mass index (FMI) [fat mass (kg)/height (m)^2^	Adult offspring BMI index and FMI index	5
Ko et al. 2014 [65]	Taiwan	Longitudinal cohort [Prospective] Taiwan Birth Cohort Study (National) 2005–2006	Mothers & **Fathers** of infants **(*N* = 21,248)**	Maternal report at baseline interview 6 months postpartum	*Smoking—*Preconception tobacco being before pregnancy and up to four months postpartum	1) Maternal age, 2) Nationality, 3) Education, 4) Parity, 5) Total weight gain during pregnancy, 6) Infant gender, 7) Multifetus, 8) Paternal smoking in the same period	**1)** Low Birth weight (LBW) < 2,500 g, **2)** Small for gestational age (SGA)—Birth below the 10th percentile of gender-specific birth weight for gestational age based on the 1998–2002 nationwide percentiles & **3)** Preterm birth < 37 weeks	Offspring birthweight & incidence of preterm delivery	5
Milne et al. 2013 [27]	Australia	Case–control [Retrospective] The Australian Study of Childhood Brain Tumors (Aus-CBT) (Population-based) 2005–2010	Mothers and Fathers (*N* = 1048) of children with childhood malignancy and brain tumors (CBT) (*n* = 247) & controls (*n* = 801)	Paternal self-report on mailed questionnaire	*Smoking—*Average number of cigarettes smoked per day in each calendar year from age 15 until year after child’s birth	1)Child’s ethnicity, 2) year of birth group, 3) Mother’s age group, 4) Father’s age group, 5) alcohol consumption during pregnancy, 6) household income	Diagnosis at one of 10 Australian paediatric oncology centres	Childhood brain tumors (CBT)	5
Moss et al. 2015^a^ [42]	USA	Longitudinal cohort [Prospective] National Longitudinal Study of Adolescent Health (Add Health) 1994–2008	Adolescents (grades 7 -12) followed into adulthood becoming Mothers & **Fathers** of infants** (*N* = 372**)	Paternal self-report of health behaviours at wave III interview	*Smoking—*At least one cigarette per day over the last 30 days	1) Parents age at birth, 2) Race/ethnicity, 3) Immigrant status, 4) Education level, 5) Socioeconomic status, 6) Infant sex, 7) Initiation of prenatal care, 8) Parity, 9) Time between wave III interview and conception, 10) Relationship type at wave III	Respondent self-report on Wave IV questionnaire	Gestational age & offspring birthweight	7
Mutsaerts et al. 2014^a^ [43]	The Netherlands	Cohort [Prospective] Groningen Expert Center for Kids with Obesity [GECKO] Drenthe cohort (Population-based) 2006–2007	Pregnant couples & their expectant children** (*N* = 2,264**)	Paternal self-report on baseline questionnaire during third trimester or within 6 months following delivery	*Smoking*- cigarettes per day in the 6 months prior to conception and up to delivery	Nil	Questionnaire, shortly after birth, completed by midwife or gynaecologist	Spontaneous preterm birth & Small for gestational age (SGA)	3
Northstone et al. 2014 [66]	UK	Cohort [Prospective] The Avon Longitudinal Study of Parents and Children (ALSPAC) 1991–1992	Pregnant couples where **fathers** identified as smoking regularly (*n* = 5,376) including before 11 years old **(*N* = 166)**	Paternal self-report on baseline questionnaire completed during pregnancy	*Smoking—*Prepubertal tobacco before 11 years of age	1) Parity of the mother at the time of birth of the offspring (primiparae vs multiparae), 2) Highest maternal education level 3) Housing tenure 4) Maternal smoking during pregnancy 5) Paternal smoking at conception	**1)** BMI, **2)** Waist circumference, **3)** Total-body fat mass, & **4)** Lean mass	Offspring adiposity	7
Orsi et al. 2015 [67]	France	Case–Control [Retrospective] ESTELLE study (Population-based) 2010—2011	Mothers and fathers **(*N* = 247)** of offspring suffering childhood acute leukemia (CL) (*N* = 69) [Cases] & cancer free age matched offspring (*N* = 178) [Controls]	Paternal self-report on baseline questionnaire	*Smoking –*Tobacco smoking during the 3-month period preceding conception; the “pre-conception period”	1)Offspring Age 2) Offspring Sex 3) Mother’s age at child’s birth 4) Mother’s education 5) Birth order	Diagnosed with CL < 15 years old as per the National Registry of Childhood Hematopoietic Malignancies (NRCH) criteria	Childhood acute leukemia (CL)	7
Sapra et al. 2016 [68]	USA	Cohort [Prospective] Longitudinal Investigation of Fertility and the Environment [LIFE]) 2005–2009	Couples attempting pregnancy **(Males [*N* = 501])**	Paternal self-report at baseline interview	*Smoking—*Lifetime exposure to tobacco products (including cigarettes, electronic cigarettes, cigars, pipes, waterpipes, chewing tobacco, snuff and dip)	1) Race/ethnicity, 2) Education, 3) Income, 4) Age, 5) Alcohol use, 6) Caffeine use, 7) BMI, 8) Blood cadmium in each partner, 9) Couple's mean age, 10) Difference in partners' ages	Time to pregnancy (TTP) in menstrual cycles	Pregnancy	7
Svanes et al. 2017 [69]	**Northern Europe** Norway, Sweden, Iceland, Denmark, Estonia	Combined Cohort [Prospective] European Community Respiratory Health Survey (ECRHS) (1989–1992) & Respiratory Heath in Northern Europe (RHINE) (Population-based) 1991—2012	Mothers and **Fathers (*N*** **= 3,777)** of offspring aged 2–51 years (*n* = 24,168)	Paternal self-report on RHINE III questionnaire	*Smoking –* Tobacco smoking prior to conception including period around birth	1)Age 2) Study centre 3) Parental age 4) Parental asthma before age 10, 5) Parental education	Diagnosis via parental report	Offspring asthma before/after 10 years	6
Wang et al. 2022 [70]	China	Cohort [Retrospective] National Free Pre-Pregnancy Checkups Project (NFPCP) (Population-based) 2010–2016	Non-smoking women and their smoking husbands** (*N* = 190,529)**	Paternal self-report at preconception health examination	*Smoking—*Tobacco while attempting conception in the following 6 months	1) Maternal and paternal age at last menstrual period, 2) Higher education, 3) Han ethnicity, 4) Preconception body mass index (BMI), 5) Alcohol drinking, 6) Parental passive smoking, 7) History of adverse pregnancy outcomes, 8) Region of the service station	Delivery before 37 completed gestational weeks	Preterm birth (PTB)	5
Wang et al. 2018 [71]	China	Cohort [Retrospective] National Free Pre-Pregnancy Checkups Project (NFPCP) (Population-based) 2010–2016	Non-smoking women and their **husbands (*N* = 5,770, 691)**	Paternal self-report at preconception health examination	*Smoking—*Tobacco while attempting conception in the following 6 months	1) Maternal and paternal age at last menstrual period, 2) Higher education, 3) Han ethnicity, 4) Preconception body mass index (BMI), 5) Alcohol drinking, 6) Parental passive smoking, 7) History of adverse pregnancy outcomes, 8) Region of the service station	Fetal death before week 28 of gestation	Spontaneous abortion (SA)	6
Wesselink et al. 2019 [72]	USA	Cohort [Prospective] Preconception pregnancy planner cohort study online (PRESTO) 2013–2018	Couples attempting pregnancy **(Males** ***N*** **= 1,411)**	Paternal self-report on baseline questionnaire	*Smoking—*Tobacco while attempting conception for ≤ 6 menstrual cycles	1) Age, 2) Race/ethnicity, 3) Education, 4) Annual household income, 5) BMI, 6) Sugar sweetened beverage intake, 7) Healthy eating index score, 8) Multivitamin or folic acid supplement use, 9) Sleep duration, 10) PSS-10 score, 11) MDI score, 12) Parity, 13) Intercourse frequency, 14) Doing something to improve chances of conception	Pregnancy attempt time = (Menstrual cycles of attempt time at baseline) + [(Last menstrual period [LMP] date from most recent followup questionnaire—date of baseline questionnaire)/Cycle length] + 1	Fecundability	5
You et al. 2022 [73]	China	Cohort [Prospective] Children lifeway Cohort 2018 -	Mothers and **Fathers (*N* = 1,037)** of first grade students (7–8 years old)	Paternal self-report at baseline interview	*Smoking—*Tobacco smoking before conception	1)Sex 2) Actual age 3) Father overweight, 4) Mother overweight 5) Percentage of food expenditure 6) Educational level of parents 7) Caesarean Sect. 8) Birthweight 9) Breastfeeding 10) Other household smoking 11) Mother exposed to SHS during pregnancy 12) Picky eaters 13) TV watching time 14) physical exercise 15) Frequency of eating fried/baked food 16) Late-night dinners 17) Vegetable and fruit 18) Snack consumption	Age and sex specific BMI cut-off points according to the growth standard of China “Screening for overweight and obesity among school-age children and adolescents”	Offspring overweight/obesity	7
Zhou et al. 2020 [74]	China	Cohort [Prospective] National Preconception Health Care Project (NPHCP) (Population-based) 2010–2012 * with matched case control	Couples attempting pregnancy **(Males [*N* = 566,439])**	Paternal self-report at baseline interview	*Smoking—*Tobacco smoking before conception	1) Maternal age, 2) Education, 3) Occupation, 4) Residence status, 5) Self-reported medical history, 6) Smoking, 7) Second hand smoking, 8) Alcohol consumption, 9) Folic acid supplement, 10) Paternal alcohol consumption	[*Primary] Birth defects = diagnosis on hospital records of first 42 days after delivery [*Secondary] Birth defect types = congenital heart disease, limb anomalies, clefts, digestive tract anomalies, gastroschisis and neural tube defects	Offspring birth defects	7
Zwink et al. 2016 [75]	Germany	Case–control [Retrospective] (Population based) 2009-Ongoing	Mothers **& Fathers** of cases **(*N* = 158**) and controls **(*N* = 474)**	Maternal report on baseline interview at approximately 8 years postpartum	*Smoking* -"Periconceptional" tobacco being 3 months before conception until the fourth month of pregnancy	1) Gender, 2) Birth year of the child, 3) Maternal age, 4) BMI, 5) Maternal body weight	Diagnosis of **1)** Esophageal atresia with or without tracheoesophageal fistula (EA/TEF) or **2)** Anorectal malformations (ARM) ARM's	Offspring malformations	4
**Stress**									
Bae et al. 2017 [76]	USA	Cohort [Prospective] Longitudinal Investigation of Fertility and the Environment [LIFE]) (Population-based) 2005–2009	Couples attempting pregnancy and their expectant children **(*N*** **= 235)**	Paternal self-report at baseline interview assessed by Cohen's Perceived Stress Scale [PSS-4]	*Stress*—& lifetime history of physician-diagnosed anxiety and/or mood disorders	1) Age, 2) Serum cotinine, 3) Annual income, 4) Maternal parity	Secondary sex ratio (SSR) [Males:Females at birth]	Offspring sex	6
Mutsaerts et al. 2014^a^ [43]	The Netherlands	Cohort [Prospective] Groningen Expert Center for Kids with Obesity [GECKO] Drenthe cohort (Population-based) 2006–2007	Pregnant couples & their expectant children** (*N* = 2,264**)	Paternal self-report on baseline questionnaire during third trimester or within 6 months following delivery	*Stress—*Paid working hours < 16 h per week	Nil	Questionnaire, shortly after birth, completed by midwife or gynaecologist	Spontaneous preterm birth & Small for gestational age (SGA)	3
Wesselink et al. 2018 [77]	USA	Cohort [Prospective] Preconception pregnancy planner cohort study online (PRESTO) 2013–2018	Couples attempting pregnancy **(Males *N* = 1,272)**	Paternal self-report on baseline questionnaire assessed by the Perceived stress scale [PSS]	*Stress—*Perceived stress in the last month	1) Age, 2) BMI, 3) Race/ethnicity, 4) Education, 5) Household income, 6) Employment status, 7) Work duration, 8) Physical activity	Pregnancy attempt time = (Menstrual cycles of attempt time at baseline) + [(Last menstrual period [LMP] date from most recent followup questionnaire—date of baseline questionnaire)/Cycle length] + 1	Fecundability	7
**Nutrition**									
Bailey et al. 2014 [29]	Australia	Case–control [Prospective] The Australian Study of Causes of acute lymphoblastic leukemia (ALL) in children (Aus-ALL). (Population-based) 2003–2007	Mothers and Fathers of children with ALL (*n* = 285) and controls (*n* = 595)	Paternal self-report on food frequency questionnaire (FFQ)	*Folate & Vitamins B6/B12—*during the 6 months before conception	1)Birth order 2) best parental education, 3) paternal age, 4) paternal smoking in the conception year, 5) year of agreement and FFQ version, 6) supplement use (folate, B6, or B12), 7) control state, 8) control sex, 9) control age	Diagnosis at one of 10 Australian paediatric oncology centres	Childhood acute lymphoblastic leukemia (ALL)	5
Greenop et al. 2015 [28]	Australia	Case–control [Retrospective] The Australian Study of Childhood Brain Tumors (Aus-CBT) (Population-based) 2005–2010	Mothers and Fathers (*N* = 866) of children with childhood malignancy and brain tumors (CBT) (*n* = 237) & controls (*n* = 629)	Paternal self-report on food frequency questionnaire (FFQ)	*Folate & Vitamins B6/B12—*during the 6 months before conception	1)Control age, 2) control sex, 3) control state of residence, 4) child’s year of diagnosis/recruitment, 5) paternal age, 6) best parental education, 7) child’s ethnicity, 8) paternal preconceptional high alcohol consumption	Diagnosis at one of 10 Australian paediatric oncology centres	Childhood brain tumors (CBT)	5
Hatch et al. 2018 [78]	USA	Cohort [Prospective] Preconception pregnancy planner cohort study online (PRESTO) 2013–2017	Couples attempting pregnancy **(Males *N* = 1,045)**	Paternal self-report on food frequency questionnaire (FFQ) at baseline	*Sugar sweetened beverage intake –*Servings per week in the past month	1)Male and female age, 2) male and female BMI, 3) age, 4) race/ethnicity, 5) education, 6) annual household income, 7) smoking history, 8) BMI, 9) physical activity, 10) caffeine intake, 11) alcohol intake, 12) sleep duration, 13) perceived stress scale score, 14) intercourse frequency	Time to pregnancy (TTP) [(menstrual cycles of attempt time at baseline) + [(LMP date from most recent follow-up questionnaire—date of baseline questionnaire)/cycle length] + 1]	Fecundability	6
Hoek et al. 2019 [79]	The Netherlands	Cohort [Prospective] Rotterdam Periconception Cohort (PREDICT Study) (Hospital-based) 2010–2015	Pregnant couples **(*N* = 511)** producing spontaneous pregnancy (*n* = 303) or IVF/ICSI pregnancy (*n* = 208)	Paternal self-report on baseline questionnaire	*Folate—*"Periconceptional" status being 14 weeks before pregnancy and up to 10 weeks of gestation	1) Gestational age at the time of ultrasound, 2) Paternal age, 3) Paternal smoking and alcohol, 4) Geographic origin, 5) Maternal age, 6) Maternal BMI, 7) Maternal smoking and alcohol, 8) Parity, 9) RBC folate levels, 10) Education level, 11) Geographic origin, 12) Fetal gender	**1)** Crown-rump length (CRL) & **2)** Embryonic volume (EV) at 7, 9 and 11 weeks of gestation	Embryonic growth trajectories	7
Lippevelde et al. 2020 [80]	Norway	Combined cohort [Prospective] Young-Health Study in Nord-Trondelag (Young-HUNT 1 1995–1997 & Young-HUNT 3 2006–2008)	Adolescents (13–19 years old) followed into adulthood becoming Mothers & Fathers of infants. Young-HUNT 1 **Father**-offspring dyads (***N* = 2,140**). Young-HUNT 3 **Father**-offspring dyads **(*N* = 391**)	Adolescent self-report on baseline questionnaire	*Diet*—Dietary exposures during adolescence	**1)** Adolescents age, **2)** BMI z-score **3)** Education plans **4)** Chewing tobacco use **5)** Smoking **6)** Alcohol use	**1)** Birthweight (g) **2)** Length (cm) **3)** Head circumference (cm) **4)** Placenta weight (g), **5)** Gestational length (weeks) & **6)** Ponderal index—Adiposity ([Birthweight (g) /Birth length^3^ (cm)]*100)	Neonatal health of offspring	8
Martin-Calvo et al. 2019 [81]	USA	Cohort [Prospective] Environment and Reproductive Health Study [EARTH] 2007–2017	Subfertile couples undergoing fertility treatment **(Males** ***N *= 108)** producing singletons [*n* = 85), twins (*n* = 54) & triplets [*n* = 3])	Paternal self-report on baseline food frequency questionnaire (FFQ)	*Folate—*Preconception intake prior to or up to 12 weeks after the day of peak oestradiol concentration during a fertility treatment cycle (IVF/ICSI/IUI)	1) Age, 2) Choline, betaine, methionine, vitamin B6, vitamin B12, 3) Total energy intake, 4) Diet quality, 5) Maternal BMI, 6) Maternal smoking status, 7) Infertility diagnosis, 8) Type of fertility treatment	**1)** Gestational age at delivery (days), **2)** Live birth of a neonate ≥ 24 weeks of gestation, & **3)** Gestational age-adjusted birthweight	IVF/ICSI/IUI induced live birth	7
Mitsunami et al. 2021 [82]	USA	Cohort [Prospective] Environment and Reproductive Health Study [EARTH] 2007–2018	Subfertile couples (**Males [*N*** **= 231])** undergoing IVF cycles (*n* = 407)	Paternal self-report on baseline food frequency questionnaire (FFQ)	*Diet—*patterns 1 (processed foods) & 2 (whole/unprocessed foods) over the previous 12 months	1) Men's age, 2) Total caloric intake, 3) BMI, 4) Race, 5) Smoking status, 6) Education level, 7) Physical activity, 8) Women's age + BMI, 9) Couple's primary infertility diagnosis, 10) Treatment protocol, 11) Women's adherence to the two dietary patterns, 12) Women's race, 13) Women's smoking status	**1)** Fertilization rate, **2)** Probability of implantation, **3)** Clinical pregnancy, & **4)** Probability of live birth per initiated treatment cycle	IVF/ICSI induced live birth	7
Moss et al. 2015^a^ [42]	USA	Longitudinal cohort [Prospective] National Longitudinal Study of Adolescent Health (Add Health) 1994–2008	Adolescents (grades 7 -12) followed into adulthood becoming Mothers & **Fathers** of infants** (*N* = 372**)	Paternal self-report of health behaviours at wave III interview	*Diet—*Fast food consumption	1) Parents age at birth, 2) Race/ethnicity, 3) Immigrant status, 4) Education level, 5) Socioeconomic status, 6) Infant sex, 7) Initiation of prenatal care, 8) Parity, 9) Time between wave III interview and conception, 10) Relationship type at wave III	Respondent self-report on Wave IV questionnaire	Gestational age & offspring birthweight	7
Oostingh et al. 2019 [83]	The Netherlands	Cohort [Prospective] Rotterdam Periconception Cohort (PREDICT Study) (Hospital-based) 2010–2016	Pregnant couples **(Males [*N*** **= 638])**	Paternal self-report on baseline food frequency questionnaire (FFQ) before 8 weeks of gestation	*Diet—*Habitual food intake and dietary patterns in a four week period during periconception being 14 weeks before and up to 10 weeks following conception	1) Gestational age, 2) Maternal and paternal total energy intake, 3) Maternal and paternal BMI, 4) Maternal age, 5) Maternal and paternal smoking, 6) Nulliparous, 7) Fetal gender	**1)** Longitudinal crown-rump length (CRL), & **2)** Embryonic volume (EV), via transvaginal ultrasound, at 7, 9 and 11 weeks of gestation	First trimester embryonic growth	6
Twigt et al. 2012 [84]	The Netherlands	Cohort [Prospective] ‘Achieving a Healthy Pregnancy’ (AHP) (Hospital-based) 2007–2010	Subfertile couples **(Males [*N* = 199])** with IVF treatment and embryo transfer within 6 months after AHP	Paternal self-report on baseline questionnaire	*Diet –*Main food groups 1)Whole wheat 2) Unsaturated oils 3) Vegetables 4) Fruits 5) Meat 6) Fish	1)Maternal age 2) Maternal smoking 3)Preconception Dietary Risk Score [PDR] of the partner 4) Maternal and Paternal BMI	A pregnancy with positive fetal heart action at around 10 weeks after embryo transfer confirmed by ultrasonography	IVF/ICSI induced ongoing pregnancy	5
Wesselink et al. 2016 [85]	USA	Cohort [Prospective] Preconception pregnancy planner cohort study online (PRESTO) 2013–2017	Couples attempting pregnancy **(Males *N* = 662)**	Paternal self-report on food frequency questionnaire (FFQ) at baseline	*Diet –* Caffeinated beverages; approximate servings per week	1) Age, 2) race/ethnicity, 3) education, 4) BMI, 5) smoking history, 6) alcohol intake, 7) intercourse frequency, 8) sleep duration, 9) work time	Time to pregnancy (TTP) [(menstrual cycles of attempt time at baseline) + [(LMP date from most recent follow-up questionnaire—date of baseline questionnaire)/cycle length] + 1]	Fecundability	6
Xia et al. 2016 [86]	USA	Cohort [Prospective] Environment and Reproductive Health Study [EARTH] 2007–2014	Subfertile couples (**Males [*N*** **= 142])** undergoing IVF/ICSI cycles (*n* = 248)	Paternal self-report on baseline food frequency questionnaire (FFQ)	*Diet—*Dairy intake in the previous 12 months	1) Age, 2) BMI, 3) Smoking status, 4) Total exercise time, 5) Dietary patterns, 6) Alcohol, 7) Caffeine, 8) Total energy intake, 9) Female dairy intake, 10) Female age, 11) Prudent dietary pattern, 12) Western dietary pattern	**1)** Fertilization rate, **2)** Implantation rate, **3)** Clinical pregnancy rate & **4)** Live birth rate per initiated cycle	IVF/ICSI induced live birth	7
Xia et al. 2015 [87]	USA	Cohort [Prospective] Environment and Reproductive Health Study [EARTH] 2007–2014	Subfertile couples (**Males [*N* = 141])** undergoing IVF/ICSI cycles (*n* = 246)	Paternal self-report on baseline food frequency questionnaire (FFQ)	*Diet—*Meat intake in the previous 12 months	1) Age, 2) Total energy intake, 3) BMI, 4) Alcohol, 5) Caffeine, 6) Prudent dietary pattern, 7) Western dietary pattern, 8) Infertility diagnoses, 9) Mode of insemination, 10) Female meat intake	**1)** Fertilization rate, **2)** Implantation rate, **3)** Clinical pregnancy rate & **4)** Live birth rate per initiated cycle	IVF/ICSI induced live birth	7

^a^ Studies covered in multiple exposure sections

^b^Total scores from quality assessment using the Newcastle–Ottawa Scale

**Table 3:** Summary table of findings from included studies

First Author & Year	Results from paternal exposure	Quality score ±
**Body composition**		
Bowatte et al. 2022 [25]	Both ever asthma risk in offspring and asthma before age 10 years old were associated with father’s high BMI trajectory (relative risk ratio [RRR] = 1.72 [95% CI: 1.00, 2.97] and RRR = 1.70 [95% CI: 0.98, 2.93], respectively). In the sex-stratified analysis, only the high BMI trajectory of fathers was associated with offspring ever allergic asthma (RRR = 2.04 [95% CI: 1.12, 3.72]; *P* = 0.02)	5
Broadney et al. 2017 [35]	Paternal pre-pregnancy body mass index [BMI] categories overweight [25.0—29.9 kg/m^2^], obese class I [30.0—34.9 kg/m^2^], and obese class II/III [> 35 kg/m^2^] are associated with reduced neonatal IgM levels (β = -0.08, [95% CI: -0.13, -0.03], *P* = 0.001); (β = -0.07, [95% CI: -0.13, -0.01], *P* = 0.029]); (β = -0.11, [95% CI: -0.19, -0.04], *P* = 0.003). Paternal overweight or obesity (class I or II/III) is not associated with the neonatal inflammation score (β = 0.003, [95% CI: -0.10, 0.11]); (β = 0.05, [95% CI: -0.07, 0.17]); (β = 0.07, [95% CI: -0.09, 0.23]) or CRP level (β = 0.02, [95% CI: -0.04, 0.09]); (β = 0.01, [95% CI: -0.07, 0.09]); (β = 0.004, [95% CI: -0.10, 0.10])	6
Casas et al. 2017 [36]	Zero association identified between paternal pre-pregnancy underweight [< 18.5 kg/m^2^] or obese fathers [≥ 30 kg/m^2^] and cognitive and psychomotor scores; Global cognitive index (β = 2.78, [95% CI: -8.40, 13.97]), (β = 0.51, [95% CI: -1.68, 2.69]); Memory (β = 4.63, [95% CI: -7.04, 16.31]), (β = 1.67, [95% CI: -0.62, 3.95]); Motor (β = -5.42, [95% CI: -17.51, 6.67]), (β = -0.96, [95% CI: -3.35, 1.42]). There is also no association between behavioural outcomes at pre-school age and underweight or obese fathers; ADHD Inattention (IRR = 3.46, [95% CI: 0.77, 15.49]), (IRR = 2.12, (95% CI: 0.73, 6.17); Hyperactivity (IRR = 1.38, [95% CI: 0.39, 4.76]), (IRR = 1.38, [95% CI: 0.96, 1.99]); Childhood Asperger Syndrome Test [CAST] (IRR = 0.85, [95% CI: 0.50, 1.46]), (IRR = 1.01, [95% CI: 0.91, 1.13])	9
Chen et al. 2021 [37]	The birth defect rate was significantly higher when paternal prepregnancy BMI ≥ 25 kg/m2 in IVF cycles (aOR 1.82, 95% CI: 1.06,3.10). Couples with paternal prepregnancy BMI ≥ 25 kg/m2 had a four-fold increased risk of congenital malformations of the musculoskeletal system (aOR 4.38, 95% CI: 1.31,14.65) *P* = 0.017 compared to couples with paternal prepregnancy BMI < 25 kg/m2. This association still remained after adjustment for confounding factors (aOR 4.55, 95% CI 1.32–15.71). No association was seen between paternal prepregnancy BMI and risk of other subcategories of birth defects	5
Fang et al. 2020 [38]	Pre-pregnancy BMI was roughly associated with TTP among men with BMI ≥ 24 (FOR 0.97 95%CI: 0.95,0.99); however, this association for men disappeared after adjusting for demographic characteristics (aFOR 1.01 95%CI: 0.98,1.02). Following logistic regression, no association was observed between male pre-pregnancy BMI ≥ 24 and subfecundity (aOR 0.97 95%CI: 0.92 – 1.03)	5
Fleten et al. 2012 [39]	Using absolute BMI values, paternal pre-pregnancy BMI and offspring BMI at age 3 years are associated (β = 0.038, [95% CI: 0.033, 0.044], *P* = 0.018). Using BMI as z-score [standard deviation] (β = 0.125, [95% CI: 0.107, 0.143], *P* = 0.805), there is no longer an association	6
Guo et al. 2022 [40]	Following multivariate adjustment, husbands who were underweight had significantly higher risk (OR = 1·17 [95% CI: (1·15, 1·19)] of SGA compared with the husband with normal BMI. In addition, a significant and increased risk of LGA was observed for overweight and obese men (OR = 1·08 [95% CI: 1·06,1·09]); (OR = 1·19 (95% CI: 1·17, 1·20)] respectively. Reduced paternal BMI was associated with an increased risk of SGA when paternal BMI was less than 22·64 (P non-linear < 0·001). Meanwhile, increasing paternal BMI were associated with an increased risk of LGA when paternal BMI was more than 22·92 (*P* non-linear < 0·001)	6
Hoek et al. 2022 [41]	Paternal periconceptional BMI is negatively associated with the fertilization rate (β = − 0.01, [SE = 0.004], *P* = 0.002]); for every increase in paternal BMI point the fertilization rate decreased 1%. Paternal BMI is not associated with the TMSC (β = − 2.48, [SE = 1.53], *P* = 0.11]), the KIDScore (β = − 0.01, [SE = 0.02], *P* = 0.62]), the embryo usage rate (β = − 0.001, [SE = 0.004], *P* = 0.84]), a positive pregnancy (β = 0.03, OR = 1.03, *P* = 0.49), fetal heartbeat (β = 0.03, OR = 1.03, *P* = 0.51) or live birth (β = 0.01, OR = 1.01, *P* = 0.82)	8
Johannessen et al. 2020 [33]	Among offspring with ECRHS/RHINE fathers who had become overweight during puberty, there was an increased risk of adult offspring’s asthma without nasal allergies (RRR = 2.36 [95% CI: 1.27, 4.38]), compared with fathers who had never been overweight. Offspring’s overweight status at age 8 years was positively associated with adult offspring’s asthma without nasal allergies (RRR = 1.50 [95% CI: 1.05, 2.16]. The risk of offspring’s overweight status at age 8 years was greater if the father was overweight at the same period [OR = 2.23 [95% CI: 1.45, 3.42] compared with the offspring having fathers who had never been overweight	6
Lonnebotn et al. 2022 [34]	Fathers’ overweight before puberty had a negative indirect effect, mediated through sons’ height, on sons’ forced expiratory volume in one second (FEV_1_) (beta (95% CI): − 144 (− 272, − 23) mL) and forced vital capacity (FVC) (beta (95% CI): − 210 (− 380, − 34) mL), and a negative direct effect on sons’ FVC (beta (95% CI): − 262 (− 501, − 9) mL); statistically significant effects on FEV_1_/FVC were not observed	7
Moss et al. 2015^a^ [42]	Paternal preconception overweight [25.0—29.9 kg/m^2^] and obesity [> 30 kg/m^2^] is not associated with gestational age (-0.19, [95% CI: -1.30, 0.91], *P* = 0.37); (-0.39, [95% CI: -1.71, 0.94], *P* = 0.28), or offspring birthweight (35.6, [95% CI: -1.40, 211.3], *P* = 0.34); (76.8, [95% CI: -74.6, 228.1], *P* = 0.16)	7
Mutsaerts et al. 2014^a^ [43]	No association identified between paternal pre-pregnancy BMI and spontaneous preterm birth (OR = 0.99, [95% CI: 0.93, 1.06]) or SGA (0.96, [95% CI: 0.91, 1.01])	3
Noor et al. 2019 [44]	Cord blood DNA methylation at 9 CpG sites is associated with paternal BMI independent of maternal BMI (*P* = < 0.05). Methylation at cg04763273, between TFAP2C and BMP7, decreased by 5% in cord blood with every 1-unit increase in paternal BMI (*P* = 3.13 × 10 -҆ꝰ), decreases persist at ages 3 (*P* = 0.002) and 7 (*P* = 0.004). Paternal BMI is associated with methylation at cg01029450 in the promoter region of the ARFGAP3 gene; methylation at this site is also associated with lower infant birthweight (β = − 0.0003; SD = 0.0001; *P* = 0.03)	7
Pomeroy et al. 2015 [23]	Paternal pre-pregnancy BMI is positively associated with neonatal neck-rump length (β = 0.12, *P* = 0.008) and the distal limb segments [lower arm/lower leg length] (β = 0.09, *P* = 0.006);(β = 0.09, *P* = 0.003). Neonatal birthweight (β = 0.08, *P* = 0.003), proximal limb segments [upper arm/thigh length] (β = 0.10, *P* = 0.001);(β = 0.08, *P* = 0.008), relative upper limb length (β = 0.10, *P* = 0.002) and relative lower limb length (β = 0.09, *P* = 0.004) are associated with paternal height only. Neonatal head circumference and adiposity are only associated with maternal pre-pregnancy height and BMI	6
Retnakaran et al. 2021 [45]	Offspring birthweight increases by 10.7 g per unit increase in paternal pregravid BMI ([95% CI: 0.5, 20.9], *P* = 0.04), yet paternal pregravid BMI is not an independent predictor for LGA (aOR = 1.15, [95% CI: 0.92, 1.44]) or SGA (aOR = 0.88, [95% CI: 0.67, 1.17]). When modelled separately, paternal pregravid weight (*P* = 0.04), not height (*P* = 0.43), is associated with offspring birthweight	8
Robinson et al. 2020 [46]	No association identified between paternal BMI overweight [≥ 25 kg/m^2^- < 30 kg/m^2^], obese class I [≥ 30 kg/m^2^- < 35 kg/m^2^] and obese class II [≥ 35 kg/m^2^] and offspring behavioural issues or psychiatric symptoms at 7–8 years; P trend for behavioural outcomes range from 0.13 [Maternal reported ADHD diagnosis] to 0.79 [Prosocial behaviours]	7
Sun et al. 2022 [47]	Compared with normal weight men, paternal pre-pregnancy overweight was associated with a significantly increased risk of preterm birth (aOR 1.34 95% CI: 1.25,1.45) and low birth weight (aOR 1.60 95% CI: 1.46–1.74) in offspring. There was also an increased risk of preterm birth (aOR 1.26 95% CI: 1.14,1.40) and low birth weight (aOR 1.40 95% CI: 1.25,1.58) in offspring of paternal pre-pregnancy obesity	7
Sundaram et al. 2017 [48]	Male BMI [25—< 35 kg m^2^] and [≥ 35 kg m^2^] is not associated with TTP, when modelled individually; (aFOR = 0.92, [95% CI: 0.70, 1.22]), (aFOR = 0.83, [95% CI: 0.53,1.28]). Obese class II couples (BMI. > 35.0 kg/m2) associate with fecundability (aFOR = 0.41, [95% CI: 0.17, 0.98]) having a longer TTP in comparison to couples with normal BMI (< 25 kg/m2) (aFOR = 0.91, [95% CI: 0.25, 3.37])	8
Umul et al. 2015 [49]	Increasing paternal BMI is inversely associated with sperm concentration (*P* = 0.02), sperm motility (*P* = 0.04), the clinical pregnancy rate (*P* = 0.04), and the live birth rate (*P* = 0.03). Zero association identified between paternal BMI and the fertilization rate (*P* = 0.89) or the implantation rate (*P* = 0.62)	2
Wei et al. 2022 [50]	Paternal pre-pregnancy overweight and obesity are associated with a higher risk of low birth weight (LBW) (overweight: OR = 1.637, 95% CI: 1.501,1.784); (obesity: OR = 1.454, 95% CI: 1.289, 1.641) and very low birth weight (VLBW) (overweight: OR = 1.310, 95% CI: 1.097,1.564); (obesity: OR = 1.320, 95% CI: 1.037, 1.681). Paternal pre-pregnancy underweight is associated with a lower risk of LBW (OR = 0.660, 95% CI: 0.519, 0.839). Parents who were both excessive-weights in pre-pregnancy BMI, as well as overweight mothers and normal-weight fathers before pre- pregnancy, were more likely to have offspring with LBW, VLBW, and extremely low birth weight (ELBW)	6
Wei et al. 2021 [51]	Paternal pre-pregnancy BMI overweight (OW) did not present associations with newborn relative telomere length (TL) in cord blood, even following adjustments (percentage change 0.93 (95% CI: -5.59,8.14));*P* = 0.772 or stratification by newborn sex (percentage change 2.09 (95% CI: -7.53,12.72));*P* = 0.686. Analysis of the combined effects of parental weight status on newborn TL showed that TL was significantly shortened among newborns whose mothers were overweight and fathers were of healthy weight when compared with those whose mothers and fathers were both of normal weight (percentage change − 8.38 (95% CI: − 15.47, − 0.92)); *P* = 0.028	6
Xu et al. 2021 [52]	Each standard deviation (SD) increment of paternal BMI (approx 3.27 kg/m^2^) is associated with an additional 29.6 g increase of birth weight ([95% CI: 5.7, 53.5], P = 0.02). As a continuous variable, one-unit increase in paternal BMI (1.0 kg/m^2^) is associated with a 9.6 g increase of offspring birth weight ([95% CI: 2.3, 17.0], P = 0.01). The association between paternal preconception body weight and offspring’s birth weight is pronounced in male neonates and neonates with overweight mothers or mothers with excessive gestational weight gain [GWG] (*P* = < 0.05)	7
Yang et al. 2015 [53]	Fathers overweight [BMI 24.0—27.9 kg/m^2^] or obese [BMI ≥ 28.0 kg/m^2^] before pregnancy have an elevated risk of giving birth to a macrosomic infant, compared with their normal weight counterparts (aOR = 1.33, [95% CI: 1.11, 1.59]);(aOR = 1.99 [95% CI: 1.49,2.65]). Paternal pre-pregnancy weight only [≥ 75.0 kgs], not height, is associated with increased risk of macrosomia (aOR = 1.49, [95% CI: 1.16, 1.92])	6
Zalbahar et al. 2017 [24]	Overweight or obese [OW/OB] fathers [> 25 kg/m^2^] and normal weight mothers [< 25 kg/m^2^] have an increased risk of offspring OW/OB at both the 5 to 14 year plus the 14 to 21 year follow-up (aOR = 2.34, [95% CI: 1.50, 3.65]);(aOR = 2.27, [95% CI: 1.60, 3.24]). This risk increases further when both parents are OW/OB (aOR = 9.95, [95% CI: 5.60, 17.69]); (aOR = 12.47, [95% CI: 7.40, 21.03]); for every unit increase in paternal and maternal BMI z-score, offspring BMI z-score increased, on average, by between 0.15% (kg m^2^) and 0.24% (kg m^2^) throughout the 5, 14 and 21 year follow-up	5
Zhang et al. 2020 [54]	Underweight [< 18.5 kg/ m^2^] male partners prolong a couples' TTP (aFOR = 0.95, [95% CI: 0.94, 0.96]) compared to male partners with normal BMI [18.5—23.9 kg/m^2^]. A combination of normal BMI women and overweight men [24.0—28.9 kg/m^2^] have the greatest opportunity for pregnancy (aFOR = 1.03, [95% CI: 1.02, 1.03]), a combination of obese women and underweight men have the least opportunity for pregnancy (aFOR = 0.70, [95% CI: 0.65, 0.76])	9
**Alcohol**		
Luan et al. 2022 [55]	The risks of rating scores on anxious/depressed were increased by 33% (RR = 1.33 [95% CI: 1.09, 1.61]) and 37% (RR = 1.37 [95% CI: 1.02,1.84]) among girls in the exposed group at ages 4 and 6, respectively. Risks of somatic complaints were increased by 18% (RR = 1.18 [95% CI: 1.00, 1.40]) and 65% (RR 1.65,[ 95% CI: 1.14, 2.38]) among boys in the exposed group at ages 4 and 6. Also, there was the increased risks of sleep problems (RR = 1.25[95% C:I 1.00,1.55]) in girls at age 4, thought problems (RR = 1.32 [95% CI: 1.01, 1.73]) in girls at age 6, and rule-breaking behaviours (RR = 1.35 [95% CI: 1.09, 1.67]) in boys at age 6	7
Milne et al. 2013 [27]	For both ALL and CBT case/control, there was some evidence of a U-shaped relationship between the amount of alcohol fathers consumed in the 12 months before the pregnancy and risk of both cancers. The odds ratios (ORs) fell with increasing consumption, to a minimum at 14–21 standard drinks a week, ALL (OR = 0.51 [95% CI: 0.32, 0.81]);CBT (OR = 0.58 [95% CI: 0.35,0.96]), and rose to a maximum at 28 drinks a week; ALL (OR = 1.20 [95% CI: 0.79,1.83); CBT (OR = 1.53 [95% CI:0.95, 2.44]). The p values for the quadratic terms in the ALL and CBT models were 0.005 and 0.02, respectively	6
Moss et al. 2015^a^ [42]	Paternal preconception alcohol intake > once a month is not associated with offspring birthweight (− 85.9, [95% CI: -336.2, 164.3], *P* = 0.50) or offspring gestational age (− 0.10, [95% CI: -0.96, 0.77], *P* = 0.83)	7
Mutsaerts et al. 2014^a^ [43]	Paternal preconception alcohol intake > 7 units/week is not associated with spontaneous preterm birth (OR = 1.08, [95% CI: 0.64, 1.83]) or SGA (OR = 1.07, [95% CI: 0.73, 1.56])	3
Xia et al. 2018 [56]	In the paternal alcohol-exposed group [> 81 g/wk], male offspring have shorter mean AGDs; for AGD-AP at birth (β =—1.73, P = 0.04) and 12 months (β = -7.29, *P* = 0.05), and shorter mean AGD-AS at 6 months (β =—4.91, *P* = 0.02). Female offspring have shorter mean AGD-AF (β = -0.72, *P* = 0.02) at birth yet longer mean AGD AC (β = 2.81, *P* = 0.04) and AGD-AF (B = 1.91, *P* = 0.04) at 12 months	8
Zuccolo et al. 2016 [57]	Increased odds of microcephaly at birth with alcohol dose per occasion at 5 + units/sitting; [1—2 units] (OR = 1.48, [95% CI: 0.77, 2.84], *P* = 0.238), [3–4 units] (OR = 1.64, [95% CI: 0.85, 3.16], *P* = 0.140), [5 + units] (OR = 1.93, [95% CI: 1.01, 3.70], *P* = 0.048). The average paternal preconception alcohol dose per occasion and general head circumference at birth is not associated [1—2 units] (β = -0.00, [95% CI: -0.05, 0.04], *P* = 0.831), [3–4 units] (β = -0.00, [95% CI: -0.05, 0.04], *P* = 0.915), [5 + units] (β = -0.02, [95% CI: -0.07, 0.02], *P* = 0.293)	4
**Cannabis**		
Har-Gil et al. 2021 [58]	Sperm quality is associated with cannabis use (6 [1.4], *P* = 0.022), compared with non-use (6[2.2], *P* = 0.50). Sperm volume (2.69/2.5 [1.6]), IVF fertilization (53/53 [59]), the IR (*P* = 0.46) and OPR (*P* = 0.508) are not associated with male cannabis use	2
Kasman et al. 2018 [59]	Zero association identified between male cannabis use and TTP, regardless of frequency; [< 1/month] (aTR = 0.9, [95% CI: 0.7, 1.2], *P* = 0.43), [Monthly] (aTR = 0.9, [95% CI: 0.5, 1.8], *P* = 0.73), [Weekly] (aTR = 1.0, [95% CI: 0.3, 2.9], *P* = 1.00), [Daily] (aTR = 1.1, [95% CI: 0.79, 1.5], *P* = 0.65)	6
Moss et al. 2015^a^ [42]	Paternal preconception cannabis use is not associated with gestational age (0.41, [95% CI: -0.43, 1.25], *P* = 0.34) or offspring birthweight (201.9, [95% CI: -97.6, 501.3], *P* = 0.19)	7
Nassan et al. 2019 [60]	Compared to males who are past or never cannabis users, couples where the male partner is a cannabis user at enrolment (*n* = 23) have increased probability of implantation (77.9, [95% CI: 53.5, 91.5], *P* = < 0.05) and live birth (47.6, [95% CI: 32.4, 63.3], *P* = < 0.05), independent of women's cannabis use. Clinical pregnancy is not associated with male cannabis use; (60.1, [95% CI: 42.6, 75.4])	7
Wise et al. 2018 [61]	Male current cannabis users (*n* = 100) present no association between cannabis use and fecundability (aFR = 1.01, [95% CI: 0.81, 1.27]) even following stratification by intercourse frequency (aFR = 1.35, [95% CI: 0.72, 2.53]) and timing of sexual intercourse (aFR = 1.05, [95% CI: 0.76, 1.45]). Paternal cannabis use [< 1 time/week] has slightly decreased fecundability (FR = 0.87, [95% CI: 0.66, 1.15]), compared with non-current users	6
**Physical activity**		
Moss et al. 2015^a^ [42]	Zero association identified between paternal preconception bouts of physical activity per week and gestational age (0.02, [95% CI: -0.04, 0.07], *P* = 0.53) or offspring birthweight (1.7, [95% CI: -13.0, 16.4], *P* = 0.82)	7
Mutsaerts et al. 2014^a^ [43]	Paternal preconception physical activity of moderate intensity < 1 time/week is not associated with spontaneous preterm birth (OR = 0.76, [95% CI: 0.45, 1.27]) or SGA (OR = 1.33, [95% CI: 0.95, 1.87])	3
**Smoking**		
Accordini et al. 2021 [32]	Fathers’ smoking initiation in prepuberty (generation G1) had a negative direct effect on their own FEV1/FVC (Δz-score − 0.36, 95% CI: − 0.68, -0.04) compared with fathers’ never smoking. This exposure had a negative direct effect on both offspring’s FEV1 (− 0.36, 95% CI: − 0.63, − 0.10) and FVC (− 0.50, 95% CI: − 0.80, − 0.20) (generation G2). Fathers’ smoking initiation at later ages also had a negative direct effect on their own FEV1 (− 0.27, 95% CI: − 0.51, − 0.02) and FEV1/FVC (− 0.20, 95% CI: − 0.37, − 0.04), but no effect found on offspring’s lung function	8
Accordini et al. 2018 [31]	Fathers’ smoking before they were 15 years old were associated with asthma without nasal allergies in their offspring [relative risk ratio ((RRR) = 1.43 95% CI: 1.01, 2.01]. The risk of fathers’ asthma (generation F1) was higher if their parents (generation F0) had ever had asthma (grandmothers’ asthma: (OR = 3.08 [95% CI: 1.96,4.85]); grandfathers’ asthma: (OR = 2.38 [95% CI: 1.51, 3.75]). The risk of asthma with or without nasal allergies in offspring (generation F2) was higher if the offspring’s father had ever had asthma (RRR = 2.37 and 1.70), respectively	7
Carslake et al. 2016 [62]	Paternal smoking during pre-adolescence (< age 11) is not reliably or strongly associated with BMI among sons, with an estimated association close to zero (mean difference in kg m-2 (95% CI) was -0.18 (-1.75, 1.39) for sons aged 12 ± 19 and 0.22 (-0.53, 0.97) for all ages). Among daughters, early-onset paternal smoking was imprecisely associated with an elevated BMI (mean difference was 1.50 (0.00, 3.00) for daughters aged 12 ± 19 and 0.97 (0.06, 1.87) for all ages)	6
Deng et al. 2013 [63]	During the periconceptional period, light paternal smoking [1–9 cigarettes/day] increases the risk of isolated conotruncal heart defects (aOR = 2.23, [95% CI: 1.05, 4.73]). Medium paternal smoking [10–19 cigarettes/day] increases the risk of septal defects (aOR = 2.04, [95% CI: 1.05, 3.98]) and left ventricular outflow tract obstructions (aOR = 2.48, [95% CI: 1.04, 5.95]). Heavy paternal smoking (≥ 20 cigarettes/day) provides even greater risk of isolated conotruncal heart defects (aOR = 8.16, [95% CI: 1.13, 58.84]) and left ventricular outflow tract obstructions (aOR = 13.12, [95% CI: 2.55, 67.39]). No association identified between paternal smoking and right ventricular outflow tract obstructions; light smoking (AOR = 1.84, [95% CI 0.88, 3.85]); medium smoking (aOR = 2.04, [95% CI: 0.71, 5.89]); heavy smoking (aOR = 6.02, [95% CI: 0.98, 36.77])	8
Frederiksen et al. 2020 [64]	Nil associations identified between paternal smoking before conception and childhood ALL (OR = 1.00, 95% CI: 0.73, 1.38). Paternal smoking before conception was associated with an increased risk of childhood AML in both the crude (OR = 2.55, 95% CI: 1.25, 5.21) and adjusted models (OR = 2.51, 95% CI: 1.21, 5.17)	7
Knudsen et al. 2020 [30]	In the unadjusted analysis, father’s preconception smoking, both starting before or from age 15 years, was associated with increased offspring BMI. Following adjustments, father’s smoking onset ≥ 15 years was significantly associated with increased BMI in their adult offspring (0.551, [95% CI: 0.174, 0.929]) *P* = 0.004. Father’s preconception smoking onset ≥ 15 years was also associated with increased offspring FMI (2.590 [95% CI: 0.544, 4.63]) *P* = 0.014. Further, sons of fathers’ who started to smoke ≥ 15 years of age (interaction *p* = 0.014) had significantly higher FMI compared to sons of never smoking fathers	5
Milne et al. 2013 [27]	Paternal preconception smoking showed no association with childhood brain tumor (CBT) risk (OR = 0.99 (95% CI: 0.71, 1.38); *P* = 0.54. There was also no association evident when paternal smoking was stratified by child’s age	5
Ko et al. 2014 [65]	Paternal preconception smoking [11–20 cigarettes/day] has a negative effect on overall infant birthweight (β = -19.17 [7.74], *P* = 0.013) but is not associated with gestational age (β = -0.05 [0.028], *P* = 0.108). Paternal preconception smoking [> 20 cigarettes/day] is not associated with preterm delivery (1.07, [95% CI: 0.84, 1.35]), low birth weight (1.14, [95% CI: 0.87, 1.27]), or small for gestational age [SGA] (1.12, [95% CI: 0.90, 1.40])	5
Moss et al. 2015^a^ [42]	Paternal preconception smoking at least one cigarette/day for one month is not associated with gestational age (− 0.31, [95% CI: − 1.20, 0.59], *P* = 0.50) or offspring birthweight (− 219.6, [95% CI: − 537.0, 97.8], *P* = 0.18)	7
Mutsaerts et al. 2014^a^ [43]	Paternal smoking [1–10 cigarettes/day] or [< 10 cigarettes/day] 6 months prior to conception, is associated with an increased risk of SGA (OR = 1.69; [95% CI: 1.10, 2.59]); (OR = 2.25, [95% CI: 1.51, 3.37]) but not spontaneous preterm birth (OR = 1.34, [95% CI: 0.74, 2.41]); (OR = 1.13, 95% CI: 0.59, 2.14)	3
Northstone et al. 2014 [66]	In sons whose fathers started smoking < 11 years, mean differences in BMI, waist circumference, and fat mass all show increases in measures at ages 13, 15 and 17; at 13 years BMI (2.83, [95% CI: 1.20, 4.25]), waist circumference and fat mass (4.83, [95% CI: 0.98, 8.68], *P* = 0.014);(5.79, [95% CI: 2.67, 8.91] *P* = < 0.0001), and at 15 years BMI (2.03 [95% CI: 0.45, 3.6]), waist circumference and fat mass (4.84, [95% CI: 0.99, 8.66], *P* = 0.006); (5.50, [95% CI: 1.88, 9.30], *P* = 0.004). At 17 years there is an association with BMI (3.25 [95% CI: 1.15, 5.35]) and fat mass (10.6 [95% CI: 5.40, 15.9], *P* = < 0.0001); waist not recorded. Daughters' measurements vary with associations at ages 9 (all measurements), 11 (lean mass *P* = 0.023), 13 (waist circumference *P* = 0.004 & lean mass *P* = 0.028) and 17 (fat mass *P* = 0.012)	7
Orsi et al. 2015 [67]	Pre-conception paternal smoking was significantly associated with ALL (OR = 1.2 [95% CI: 1.1,1.5)] and AML (OR = 1.5 [95% CI: 1.0–2.3]). For ALL, the ORs were higher for smoking\10 cigarettes daily than for the highest consumption; no significant trend was evidenced. For AML, significant trends were evidenced for both periods (p trend = 0.03 and 0.02, respectively), with ORs of close to 2.0 for smoking more than 15 cigarettes daily. No joint effect of paternal and maternal smoking was detected	7
Sapra et al. 2016 [68]	Paternal cigarette smoking is associated with a longer TTP compared with never users (aFOR = 0.41, [95% CI: 0.24, 0.68]); attenuated slightly after adjusting for cadmium (aFOR = 0.44, 95% CI: 0.24, 0.79). When modelling partners together, paternal cigarette smoking remains associated with a longer TTP (aFOR = 0.46, [95% CI: 0.27, 0.79]), also attenuated after adjustment for cadmium (aFOR = 0.50, [95% CI 0.27—0.91]). Zero association identified between TTP and exposure to any other tobacco products including cigars (FOR = 0.70, [95% CI: 0.45, 1.08]) or snuff and chew tobacco (FOR = 1.17, [95% CI: 0.70, 1.95]	7
Svanes et al. 2017 [69]	Non-allergic early-onset asthma (asthma without hay fever) was more common in the offspring with fathers who smoked before conception (OR = 1.68 [95% CI: 1.18,2.41]). The risk was highest if father started smoking before age 15 years (OR = 3.24 [95% CI: 1.67,6.27]), even if he stopped more than 5 years before conception (OR = 2.68 [95% CI: 1.17, 6.13]).Both a father’s early smoking debut (*P* = 0.001) and a father’s longer smoking duration (*P* = 0.01) before conception increased non-allergic early-onset asthma in offspring, even with mutual adjustment and adjusting for number of cigarettes and years since quitting smoking. A father’s smoking debut before age 11 years (102 fathers) showed the greatest increased risk (OR = 3.95, [95% CI: 1.07,14.60]), followed by smoking debut ages 11–14 (OR = 1.75, [95% CI: 1.07,1.86]) and smoking debut after age 15 (OR = 1.37, [95% CI: 1.00,1.86]). Longer duration of smoking was also associated with an increased risk, up to 1.8-fold for those smoking for more than 10 years (OR = 1.76, [95% CI: 0.96,3.25])	6
Wang et al. 2022 [70]	Hazard ratio (HR) of preterm birth (PTB) was 1.07 (95% CI, 1.06–1.09), compared with women without preconception paternal smoking. Compared with participants without preconception paternal smoking, the fully adjusted HRs of PTB were (1.04 [95% CI: 0.99,1.08]), (1.05 [95% CI: 1.01, 1.08]), (1.06 [95% CI: 1.03, 1.09]), (1.14 [95% CI: 1.07, 1.21]) and (1.15 [95% CI: 1.11, 1.19]) for participants whose husband smoked 1–4, 5–9, 10–14, 15–19, and ≥ 20 cigarettes/day respectively (***P*** linear < 0.05)	5
Wang et al. 2018 [71]	Women with exposure to paternal preconception smoking have increased odds of SA (aOR = 1.11, [95% CI: 1.08, 1.14], *P* = < 0.01). This association is evident when smoking > 10 cigarettes/day, *P* = < 0.01; [10–14 cigarettes/day] (aOR = 1.11, [95% CI: 1.06, 1.16]), [15–19 cigarettes/day] (aOR = 1.21, [95% CI: 1.09, 1.33]) and ≥ 20 cigarettes/day (aOR = 1.23, [95% CI: 1.17, 1.30)	6
Wesselink et al. 2019 [72]	Male current regular smoking, current occasional smoking, and former smoking is not associated with fecundability (FR = 0.96, [95% CI: 0.70, 1.34]), (FR = 0.83, [95% CI: 0.61, 1.13]), (FR = 1.14, [95% CI: 0.97, 1.35])	5
You et al. 2022 [73]	For those with only preconception exposure, compared with children without paternal smoking, the risk of childhood overweight and obesity was increased (OR = 1.41 [95% CI: 1.17, 1.85]). Following further adjustments, for lifestyle and dietary factors, this effect remained statistically significant (OR = 1.54 [95% CI: 1.14, 2.08]). When stratified by sex, the effects of only preconception exposure on childhood overweight and obesity was statistically significant for only boys (*p* < 0.05)	7
Zhou et al. 2020 [74]	There is an increased risk of birth defects in the continued-smoking (OR = 1.87, [95% CI: 1.36, 2.56], *P* < 0.001) and decreased-smoking groups (OR = 1.41, [95% CI: 1.10, 1.82], *P* = 0.007). Continued paternal smoking is associated with an elevated risk of congenital heart diseases (OR = 2.51, [95% CI: 1.04, 6.05], P = 0.040), limb abnormalities (OR = 20.64, [95% CI: 6.26, 68.02], *P* < 0.001), digestive tract anomalies (OR = 3.67, [95% CI: 1.44, 9.37], P = 0.007) and neural tube defects (OR = 4.87, [95% CI: 1.66, 14.28], *P* = 0.004). There is no association between continued paternal smoking and clefts (OR 1.44, [95% CI: 0.34, 5.90], *P* = 0.625) or gastroschisis (OR = 2.63, [95% CI: 0.82, 8.40] *P* = 0.103)	7
Zwink et al. 2016 [75]	Paternal periconceptional tobacco consumption is lower in the fathers of EA/TEF patients [Any smoking] n = 20 (20%) *P* = 0.003, compared with fathers of isolated ARM patients [Any smoking] *n* = 49 (40%) *P* = 0.003	4
**Stress**		
Bae et al. 2017 [76]	There is a 76% increase in risk of fathering a male infant (RR = 1.76, [95% CI: 1.17, 2.65]) in men diagnosed with anxiety disorders compared with those not diagnosed. This association is strengthened (RR = 2.03, [95% CI: 1.46, 2.84]) when modelled jointly for the couple	6
Mutsaerts et al. 2014^a^ [43]	Paternal paid working hours < 16 h/week is not associated with spontaneous preterm birth (OR = 2.21, [95% CI: 0.78, 6.26]) or SGA (OR = 0.76, [95% CI: 0.23, 2.45])	3
Wesselink et al. 2018 [77]	Men's baseline PSS scores are not associated with fecundability; [PSS score 10—14] (FR = 0.95 [95% CI: 0.79, 1.15]), [PSS Score 15–19] (FR = 1.07 [95% CI: 0.86, 1.33]), [PSS Score 20–24] (FR = 1.02 [0.76, 1.36]), [PSS Score ≥ 25] (FR = 1.03 [0.69, 1.54])	7
**Nutrition**		
Bailey et al. 2014 [29]	No significant associations identified with paternal dietary intake of folate or vitamin B6 or vitamin B12 and risk of ALL; (OR = 1.37 95% CI: 0.78, 2.40)	5
Greenop et al. 2015 [28]	No significant associations identified between risk of childhood brain tumors (CBT) and energy adjusted dietary folate > 509.5 (mcg) (OR = 0.85 95% CI: 0.56,1.28) or energy adjusted B6 > 1.71 (mg) (OR = 0.98 95% CI: 0.66,1.47). A high B12 intake (> 5.91(mcg)) was not significantly associated with an increased risk of CBT (OR = 1.74 95% CI: 1.14, 2.66)	5
Hatch et al. 2018 [78]	Male intake of sugar-sweetened beverages is associated with reduced fecundability (aFR = 0.78 95% CI: 0.63, 0.95) for ≥ 7 sugar-sweetened beverages per week compared with none. Fecundability was further reduced among those who drank ≥ 7 servings per week of sugar-sweetened sodas (aFR = 0.67 95% CI: 0.51, 0.89). The largest reduction in fecundability was seen in men who consumed seven or more energy drinks per week (FR = 0.42; 95% CI: 0.20, 0.90). Diet sodas did not have significant association with fecundability at ≥ 7 servings per week (aFR = 0.93 95% CI: 0.71, 1.2)	6
Hoek et al. 2019 [79]	In spontaneously conceived pregnancies, there is a negative association between paternal RBC folate status and CRL trajectories, in Q2 [875–1,018 nmol/L;] (β = -0.14; [95% CI:—0.28, -0.006], *P* = 0.04) and Q4 [1,196–4,343 nmol/L] (β =—0.19, [95% CI:—0.33, -0.04], *P* = 0.012). A negative association also exists for EV trajectories in Q4 (β =—0.12, [95% CI: -0.20, -0.05], *P* = 0.001). No association identified between paternal RBC folate status and CRL or EV trajectories in IVF-ICSI pregnancies [Q4] (β = 0.03, [95% CI: -0.07, 0.13], *P* = 0.55), (β = 0.03, [95% CI: -0.03, 0.08], P = 0.32)	7
Lippevelde et al. 2020 [80]	In Young-HUNT1, an extra serving of fruit per week in the paternal diet, during adolescence, is associated with a 2.35 g increase in offspring placenta weight [95% CI: 0.284, 4.42], *P* = 0.03. A slightly shorter birth length is associated with increased paternal vegetable intake during adolescence (β = -0.048, [95% CI: -0.080, -0.016], *P* = 0.003) and a lower ponderal index is associated with paternal whole grain bread consumption (β = -0.003, [95% CI: -0.005, -0.001], *P* = 0.01). Paternal lunching regularly in adolescence is associated with an increase in offspring head circumference (β = 0.160, [95% CI: 0.001, 0.320], *P* = 0.05). Birthweight is not associated with any paternal dietary exposures; [Fruit] (β = 5.84 [95% CI: -0.983, 12.7], *P* = 0.1). These associations are not observed in Young-HUNT3	8
Martin-Calvo et al. 2019 [81]	A 400 μg/day increase in preconception paternal folate intake is associated with a 2.6-day longer gestation [95% CI: 0.8, 4.3], *P* = 0.004. This association is strongest in multifetal pregnancies (β = 10.7, [95% CI: 4.6, 16.8]). Zero association identified between paternal folate intake and gestational age-specific birthweight (β = -11.4, [95% CI: -28.2, 5.4])	7
Mitsunami et al. 2021 [82]	Paternal adherence to either dietary patterns 1 or 2 is not associated with the fertilization rate during IVF or ICSI ([Pattern 1] *P* = 0.59, [Pattern 2] *P* = 0.06), ([Pattern 1] *P* = 0.72, [Pattern 2] *P* = 0.94). Zero association identified between male dietary patterns and probabilities of implantation, clinical pregnancy, or live birth; ([Pattern 1] *P* = 0.68, [Pattern 2] *P* = 0.43), ([Pattern 1] *P* = 0.35, [Pattern 2] *P* = 0.68), ([Pattern 1] *P* = 0.53, [Pattern 2] *P* = 0.10)	7
Moss et al. 2015^a^ [42]	Males eating fast food more frequently have infants born earlier than men who eat fast-food less frequently (-0.16, [95% CI: -0.32, 0.00], *P* = 0.04). There is no association between paternal fast-food consumption and birthweight (-36.0, [95% CI: -89.8, 17.8], *P* = 0.19)	7
Oostingh et al. 2019 [83]	Zero association identified between paternal dietary patterns and CRL or EV in spontaneous pregnancies; [Whole wheat grains and vegetables] (β = -0.006 [95% CI: -0.069, 0.058]), (β = 0.001 [95% CI: -0.022, 0.021]), and in IVF/ICSI pregnancies, (β = -0.015 [95% CI: -0.061, 0.031]), (β = -0.006 [95% CI: -0.025, 0.013]), independent of maternal dietary patterns	6
Twigt et al. 2012 [84]	Paternal Preconception Dietary Risk Score [PDR] did not affect the chance of pregnancy after IVF/ICSI treatment (OR = 0.95 [95% CI: 0.48,1.86]) *P* = 0.88	5
Wesselink et al. 2016 [85]	Total caffeine intake among males was associated with fecundability for ≥ 300 mg vs. < 100 mg/day (OR = 0.72, 95% CI: 0.54, 0.96)	6
Xia et al. 2016 [86]	Men's total dairy intake is not associated with the fertilization rate [Conventional IVF] (0.75, [95% CI: 0.60, 0.86], *P* = 0.29), [ICSI] (0.72, [95% CI: 0.58, 0.82], *P* = 0.18], the implantation rate (0.58, [95% CI: 0.40, 0.74], *P* = 0.87), the clinical pregnancy rate (0.51, [95% CI: 0.34, 0.68], *P* = 0.54), or the live birth rate (0.46, [95% CI: 0.28, 0.65], *P* = 0.65)	7
Xia et al. 2015 [87]	A positive association identified between paternal poultry intake and the fertilization rate, [Model 1] *P* = 0.05, [Model 2] *P* = 0.03, [Model 3] *P* = 0.03, [Model 4] *P* = 0.04, with a 13% higher fertilization rate among men in the highest quartile of poultry intake compared with those in the lowest quartile (78% vs. 65%) [Model 4]. Men's total meat intake is not associated with the implantation rate (0.52, [95% CI: 0.37, 0.67], *P* = 0.67), clinical pregnancy rate (0.45, [95% CI: 0.32, 0.59], *P* = 0.56), or live-birth rate (0.35, [95% CI: 0.22, 0.50], *P* = 0.82)	7

^a^ Studies covered in multiple exposure sections

^b^Quality score based on assessment using Newcastle–Ottawa Scale

Study participants were diverse consisting of couples either intending pregnancy or pregnant (*n* = 25), sub-fertile and seeking fertility treatment undergoing IVF/ICSI (*n* = 11), or mothers and fathers of infants (*n* = 26). Two studies included adolescents followed into parenthood as adults [42, 80], and one study included individual respondents of a national family growth survey, actively attempting pregnancy [59].

Modifiable preconception risk factors and/or health behaviour exposures examined include paternal body composition (*n* = 25), alcohol intake (*n* = 6), cannabis use (*n* = 5), physical activity (*n* = 2), smoking (*n* = 20), stress (*n* = 3), and nutrition (*n* = 13) (including dietary folate intake and consumption of foods and dietary patterns). Two papers investigated multiple exposures [42, 43].

Outcomes examined include fecundability (*n* = 6) [38, 61, 72, 77, 78, 85], (time to) pregnancy (*n* = 4) [48, 54, 59, 68], IVF/ICSI ongoing pregnancy (*n* = 1) [84] or live birth (*n* = 7) [41, 49, 58, 60, 81, 82, 86, 87], offspring birthweight or adiposity (*n* = 10) [40, 42, 45, 47, 50, 52, 53, 62, 65, 66], including small for gestational age [SGA] [43], neonatal (*n* = 1) [23] and offspring body composition (*n* = 4) [24, 30, 39, 73]. Other outcomes examined include offspring asthma (*n* = 4) [25, 31, 33, 69] and lung function (*n* = 2) [32, 34], childhood leukemia (*n* = 4) [26, 29, 64, 67], childhood brain tumours (*n* = 2) [27, 28], and offspring behavioural issues (*n* = 2) [46, 55].

**Fig. 2:**
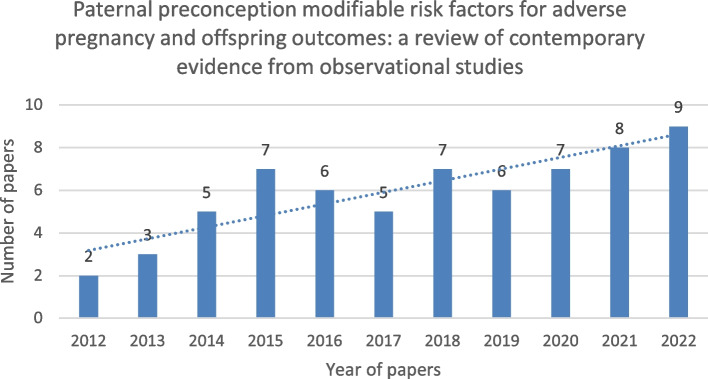
Papers included in this review

There was an increasing number of papers identified for inclusion in this review with the least number of papers published in 2012 and the greatest number of papers published in 2022 (see Fig. 2).

Results below are described for papers assessed as good quality with approximately half (*n* = 30) rated good quality and two receiving a maximum nine-star rating [36, 54] (Table 4—Newcastle Ottawa Scale [NOS] quality assessment (Cohorts)) & (Table 5—Newcastle Ottawa Scale [NOS] quality assessment (Case controls)). Results for the fair and poor-quality papers are not further described.

**Table 4:** Newcastle Ottawa Scale [NOS] quality assessment (Cohorts)

First author & Year	Newcastle Ottawa Scale—Criteria									
	**SELECTION**				**COMPARABILITY**		**OUTCOME**			**TOTAL**
	**Exposed cohort (representativeness)**	**Non-exposed cohort**	**Ascertainment of exposure**	**Outcome of interest not present at start of study**	**Based on design or analysis (AGE)**	**Based on design or analysis (OTHER FACTORS)**	**Assessment of outcome**	**Appropriate length of follow-up**	**Adequacy of follow-up of cohorts**	
** Accordini et al. 2021 **[32]	✶	✶	✶		✶	✶	✶	✶	✶	8
** Accordini et al. 2018** [31]	✶	✶	✶		✶	✶		✶	✶	7
** Bae et al. 2017** [80]		✶	✶	✶	✶	✶		✶		6
** Bowatte et al. 2022** [25]		✶	✶			✶		✶	✶	5
** Broadney et al. 2017** [76]	✶	✶			✶	✶	✶		✶	6
** Carslake et al. 2016** [62]	✶	✶				✶	✶	✶	✶	6
** Casas et al. 2017** [74]	✶	✶	✶	✶	✶	✶	✶	✶	✶	9
** Chen et al. 2021** [75]		✶	✶		✶	✶	✶			5
** Fang et al. 2020** [42]	✶	✶	✶		✶	✶				5
** Fleten et al. 2012** [67]	✶	✶		✶		✶		✶	✶	6
** Guo et al. 2022** [61]	✶	✶	✶	✶	✶	✶				6
** Har-Gil 2021** [50]		✶					✶			2
** Hatch et al. 2018** [44]		✶		✶	✶	✶		✶	✶	6
** Hoek et al. 2022** [49]		✶	✶	✶	✶	✶	✶	✶	✶	8
** Hoek et al. 2019** [88]		✶	✶		✶	✶	✶	✶	✶	7
** Johannessen et al. 2020** [33]	✶	✶			✶	✶		✶	✶	6
** Kasman et al. 2018** [37]	✶	✶	✶	✶	✶	✶				6
** Knudsen et al. 2020** [30]	✶	✶				✶		✶	✶	5
** Ko et al. 2014** [60]	✶	✶	✶		✶	✶				5
** Lippevelde et al. 2020 **[80]	✶	✶		✶	✶	✶	✶	✶	✶	8
** Lonnebotn et al. 2022** [34]	✶	✶			✶	✶	✶	✶	✶	7
** Luan et al. 2022** [73]		✶	✶	✶	✶	✶		✶	✶	7
** Martin-Calvo et al. 2019**		✶	✶	✶	✶	✶		✶	✶	7
** Mitsunami et al. 2021** [82]		✶	✶	✶	✶	✶		✶	✶	7
** Moss et al. 2015** [35]	✶	✶		✶	✶	✶		✶	✶	7
** Mutsaerts et al. 2014** [38]	✶	✶							✶	3
** Nassan et al. 2019** [52]		✶	✶	✶	✶	✶		✶	✶	7
** Noor et al. 2019** [77]		✶		✶	✶	✶	✶	✶	✶	7
** Northstone et al. 2014** [63]	✶	✶		✶		✶	✶	✶	✶	7
** Oostingh et al. 2019** [81]		✶		✶	✶	✶	✶		✶	6
** Pomeroy et al. 2015** [23]		✶			✶	✶	✶	✶	✶	6
** Retnakaran et al. 2021** [58]		✶	✶	✶	✶	✶	✶	✶	✶	8
** Robinson et al. 2020** [72]	✶	✶		✶	✶	✶		✶	✶	7
** Sapra et al. 2016** [47]		✶	✶	✶	✶	✶	✶	✶		7
** Sun et al. 2022** [66]		✶	✶	✶	✶	✶		✶	✶	7
** Sundaram et al. 2017** [45]		✶	✶	✶	✶	✶	✶	✶	✶	8
** Svanes et al. 2017** [69]	✶				✶	✶		✶	✶	6
** Twigt et al. 2012** [48]		✶		✶	✶	✶			✶	5
** Umul et al. 2015** [57]		✶					✶			2
** Wang et al. 2022** [65]	✶	✶	✶		✶	✶				5
** Wang et al. 2018** [85]	✶	✶	✶	✶	✶	✶				6
** Wei et al. 2022** [50]		✶		✶	✶	✶	✶	✶		6
** Wei et al. 2021** [51]	✶	✶	✶		✶	✶	✶			6
** Wesselink et al. 2019** [40]		✶		✶	✶	✶		✶		5
** Wesselink et al. 2018** [41]		✶	✶	✶	✶	✶		✶	✶	7
** Wesselink et al. 2016** [43]		✶		✶	✶	✶		✶	✶	6
** Wise et al. 2018** [39]		✶		✶	✶	✶		✶	✶	6
** Xia et al. 2018** [78]		✶	✶	✶	✶	✶	✶	✶	✶	8
** Xia et al. 2016** [55]		✶		✶	✶	✶	✶	✶	✶	7
** Xia et al. 2015** [56]		✶		✶	✶	✶	✶	✶	✶	7
** Xu et al. 2021** [59]		✶	✶	✶	✶	✶		✶	✶	7
** You et al. 2022** [68]		✶	✶		✶	✶	✶	✶	✶	7
** Zalbahar et al. 2017** [24]		✶			✶	✶	✶	✶		5
** Zhang et al. 2020** [46]	✶	✶	✶	✶	✶	✶	✶	✶	✶	9
** Zhou et al. 2020** [86]		✶	✶	✶	✶	✶		✶	✶	7
** Zuccolo et al. 2016** [79]	✶	✶			✶	✶				4

**Table 5:** Newcastle Ottawa Scale [NOS] quality assessment (Case controls)

Newcastle–Ottawa Critical Analysis (Case controls)—Criteria										
**First author & Year**	**SELECTION**				**COMPARABILITY**		**EXPOSURE**			**TOTAL**
	**Adequacy of case definition**	**Representativeness of the cases**	**Selection of controls**	**Definition of controls**	**Comparability of cases and controls (AGE)**	**Comparability of cases and controls (OTHER FACTORS)**	**Ascertainment of exposure**	**Same method of ascertainment for cases and controls**	**Non-Response rate**	
** Bailey et al. 2014** [29]	✶		✶		✶	✶	✶			5
** Deng et al. 2013** [83]	✶	✶		✶	✶	✶	✶	✶	✶	8
** Frederiksen et al. 2020** [70]	✶	✶	✶	✶	✶	✶		✶		7
** Greenop et al. 2015** [28]	✶		✶		✶	✶	✶			5
** Milne et al. 2013** [27]	✶	✶	✶		✶	✶		✶		6
** Milne et al. 2013** [27]	✶		✶		✶	✶	✶			5
** Orsi et al. 2015** [71]	✶	✶	✶	✶	✶	✶		✶		7
** Yang et al. 2015** [64]		✶	✶	✶	✶	✶		✶		6
** Zwink et al. 2016** [87]	✶			✶	✶	✶				4

### Body Composition

Twenty-five papers investigated associations between paternal BMI and various offspring outcomes. These papers were derived from studies (*n* = 21) conducted in the US, Europe, China, Australia, and Turkey and less than half (*n* = 11) rated as good quality.

Less than half of the papers (*n* = 10) used anthropometric assessment by the research team to determine BMI [37, 38, 40–42, 45, 47–49, 54]; heights and weights utilized to formulate BMI were determined in the preconception period, generally from males in couples undergoing IVF/ICSI [41, 49] or males in couples currently attempting pregnancy/pregnant [40, 45, 48, 54]. Of the papers validating the BMI utilizing anthropometric assessments, most were good quality and generally affirmed significant results. The remaining papers utilize retrospective reports of preconception paternal weight and height or collect paternal height and weight from medical records. Maternal reporting (*n* = 7) occurred at approximately 10 to 18 weeks gestation; or up to four months postpartum [35, 46]. Paternal self-reporting of their own weight and height (*n* = 8) occurred at approximately week 17 of gestation [39, 43, 50, 52, 53] or up to 6 months postpartum [43]. In two papers, overweight paternal status, when a child of 8 years, was reported years later through a validated drawing of silhouettes [33, 34].

The outcomes and outcome measures varied, however, ten studies assessed the association of paternal BMI with offspring BMI/bodyweight [23, 39, 40, 42, 45, 47, 50, 52, 53], and one paper assesses offspring weight and BMI changes from childhood (5 years) into adulthood (21 years) [24].

Results of associations between body composition and offspring outcomes were inconsistent. In some studies paternal preconception overweight (25.0—29.9 kg/m^2^) and obesity (> 30 kg/m^2^) were not associated with offspring birthweight [42] and paternal pregravid BMI was not an independent predictor for large for gestational age (LGA) or small for gestational age (SGA) [45]. However, other studies found that offspring birthweight increased by 10.7 g per unit increase in paternal pregravid BMI (95% CI: 0.5, 20.9, *P* = 0.04) [45], and each standard deviation (SD) increment of paternal BMI (approximately 3.27 kg/m^2^) was associated with an additional 29.6 g increase of birth weight (95% CI: 5.7, 53.5, *P* = 0.02) [52]. Further, compared with normal weight men, paternal pre-pregnancy overweight was associated with a significantly increased risk of preterm birth (aOR 1.34 95% CI: 1.25,1.45) and low birth weight (aOR 1.60 95% CI: 1.46–1.74) in offspring [47].

Paternal pregravid weight (*P* = 0.04), not height (*P* = 0.43), was associated with infant birth weight [45] and with increased risk of macrosomia (aOR = 1.49, [95% CI: 1.16, 1.92]) [53], while neonatal birth weight was associated with paternal height only (β = 0.08, *P* = 0.003) [23]. In another study, paternal pre-pregnancy BMI was only associated with offspring BMI when using absolute BMI values not BMI as a z-score [39].

Fathers’ overweight before puberty had a negative indirect effect, mediated through sons’ height, on sons’ forced expiratory volume in one second (FEV_1_) (beta (95% CI): − 144 (− 272, − 23) mL) and forced vital capacity (FVC) (beta (95% CI): − 210 (− 380, − 34) mL), and a negative direct effect on sons’ FVC (beta (95% CI): − 262 (− 501, − 9) mL) [34].

Male BMI ≥ 25 kg m^2^ was not associated with time to pregnancy (TTP) [48], yet underweight (< 18.5 kg/ m^2^) was associated with a longer TTP (adjusted fecundability odds ratio [aFOR] = 0.95, [95% CI: 0.94, 0.96]) compared to normal BMI (18.5—23.9 kg/m^2^) [54].

In couples undergoing IVF/ICSI, paternal periconceptional BMI was negatively associated with fertilization rate (β =  − 0.01 [SE = 0.004], *P* = 0.002]), while paternal BMI was not associated with the total motile sperm count (TMSC), the KIDScore, the embryo usage rate, a positive pregnancy, fetal heartbeat, or live birth [41].

Offspring methylation was associated with paternal BMI independent of maternal BMI (*P* =  < 0.05) [44]. Methylation decreased by 5% in cord blood with every 1-unit increase in paternal BMI (*P* = 3.13 × 10 -҆ꝰ), decreases persist at 3 years old (*P* = 0.002) and 7 years old (*P* = 0.004) [44]. Paternal BMI was associated with methylation at cg01029450 in the promoter region of the ARFGAP3 gene; methylation at this site was also associated with lower infant birthweight (β =  − 0.0003; SD = 0.0001; *P* = 0.03) [44].

No association was found between behavioural outcomes at pre-school age and underweight (< 18.5 kg/m^2^) or obesity (≥ 30 kg/m^2^) in fathers [36]. Equally, no associations were found between paternal BMI overweight (≥ 25 kg/m^2^- < 30 kg/m^2^), obese class I (≥ 30 kg/m^2^- < 35 kg/m^2^) and obese class II (≥ 35 kg/m^2^) and offspring behavioural issues or psychiatric symptoms at 7–8 years [46].

### Alcohol

Six papers examined alcohol as an exposure [26, 42, 43, 55–57]; three rated as good quality [42, 55, 56]. Excluding one, each paper used paternal self-reports of alcohol consumption with varying definitions; one article specified units/per week [43], the others assessed consumption more broadly either as intake ≥ 1/week [56], ≥ 1/month [42] or general intake [57]. A single study presented a maternal report of paternal preconception alcohol consumption, 3 months before conception, at 12–16 weeks gestation [55].

When examining an outcome of offspring anogenital distance (AGD), in the paternal alcohol-exposed group (> 81 g/wk), male offspring had shorter mean AGDs [56]; for AGD-AP [the centre of the anus to the cephalad insertion of the penis] at birth (β =—1.73, *P* = 0.04) and 12 months (β = -7.29, *P* = 0.05), and shorter mean AGD-AS [the centre of the anus to the posterior base of the scrotum] at 6 months (β =—4.91, *P* = 0.02) [56]. Female offspring had shorter mean AGD-AF [ the centre of the anus to the posterior convergence of the fourchette) (β = -0.72, *P* = 0.02) at birth yet longer mean AGD AC [the centre of the anus to the clitoris] (β = 2.81, *P* = 0.04) and AGD-AF (B = 1.91, *P* = 0.04) at 12 months [56]. Further, the relative risks of anxiety or depression were increased by 33% (RR = 1.33 [95% CI: 1.09, 1.61]) and 37% (RR = 1.37 [95% CI: 1.02,1.84]) among girls in the exposed group at ages 4 and 6, respectively [55]. Paternal alcohol consumption greater than once per month was not associated with offspring birthweight or gestational age [42].

### Cannabis

Paternal cannabis exposure was assessed in five papers [42, 58–61], two rate as good quality [42, 60]. Each paper has a sample size < 1,200 and each utilized paternal self-reporting of cannabis use broadly assessing general use, rather than specific amounts, over a pre-determined period (i.e., last 2 months or 12 months).

In sub-fertile couples undergoing IVF/ICSI, compared to males who were past or never cannabis users, couples where the male partner used cannabis at enrolment had increased probability of implantation (77.9, [95% CI: 53.5, 91.5], *P* =  < 0.05) and live birth (47.6, [95% CI: 32.4, 63.3], *P* =  < 0.05), independent of women's cannabis use [60]. Clinical pregnancy was not associated with male cannabis use [60], nor was gestational age or offspring birthweight [42].

### Physical activity

The associations of paternal physical activity with offspring outcomes were assessed in two papers [42, 43], one rated as good quality [42]. This study found no association between paternal preconception bouts of physical activity per week and gestational age or offspring birthweight [42].

### Smoking

The association of tobacco smoking with offspring outcomes was examined in 20 papers [27, 30–32, 42, 62–75, 88]; half (*n* = 10) were rated as good quality [31, 32, 42, 63, 64, 66–68, 73, 74] and nine papers adjusted for maternal smoking and/or paternal passive smoking in their analysis [32, 62, 64–66, 71, 73, 74].

Paternal cigarette smoking was associated with a longer TTP compared with never users (aFOR = 0.41, [95% CI: 0.24, 0.68]), while no associations were found for other tobacco products including cigars or snuff and chew tobacco [68].

Outcomes involving smoking and birth defects report that during the periconceptional period, light paternal smoking [1–9 cigarettes/day] increased the risk of isolated conotruncal heart defects (aOR = 2.23, [95% CI: 1.05, 4.73]) [63]. Medium paternal smoking [10–19 cigarettes/day] increased the risk of septal defects (aOR = 2.04, [95% CI: 1.05, 3.98]) and left ventricular outflow tract obstructions (aOR = 2.48, [95% CI: 1.04, 5.95]) [63]. Heavy paternal smoking (≥ 20 cigarettes/day) increased the risk of isolated conotruncal heart defects (aOR = 8.16, [95% CI: 1.13, 58.84]) and left ventricular outflow tract obstructions (aOR = 13.12, [95% CI: 2.55, 67.39]) [63]. Likewise, an increased risk of birth defects was found for continued-smoking (OR = 1.87, [95% CI: 1.36, 2.56], *P* < 0.001) and decreased-smoking groups (OR = 1.41, [95% CI: 1.10, 1.82], *P* = 0.007) compared with those fathers that quit smoking during early pregnancy and those who did not smoke at all during preconception [74].

Paternal preconception smoking at least one cigarette/day for one month was not associated with gestational age or offspring birthweight [42]. In contrast, a second study found sons whose fathers started smoking < 11 years, the adjusted mean differences in BMI, waist circumference, and fat mass all showed higher values at ages 13, 15, and 17 [66]. Further, the risk of childhood overweight and obesity was increased among children exposed to paternal preconception smoking compared to children without paternal smoking exposure (OR = 1.41 [95% CI: 1.17, 1.85]) [73].

Paternal preconception smoking 12 months prior to conception was associated with an increased risk of childhood acute myeloid leukemia (AML) (OR = 2.51, 95% CI: 1.21, 5.17) [64] and paternal smoking just 3 months prior to conception provided significant associations with acute lymphoblastic leukemia (ALL) (OR = 1.2 [95% CI: 1.1,1.5)] and acute myeloblastic leukemia (AML) (OR = 1.5 [95% CI: 1.0–2.3]) [67].

Paternal preconception smoking also provided significant associations with offspring lung function and asthma; fathers’ smoking initiation in prepuberty (generation G1) had a negative direct effect on their own FEV1/FVC (difference in offspring’s expected score − 0.36, 95% CI: − 0.68, -0.04) compared with fathers’ never smoking. This exposure had a negative direct effect on both offspring’s FEV1 (− 0.36, 95% CI: − 0.63, − 0.10) and FVC (− 0.50, 95% CI: − 0.80, − 0.20) (generation G2) [32]. Fathers’ smoking before age 15 years was associated with higher risk of asthma without nasal allergies in their offspring [relative risk ratio ((RRR) = 1.43 95% CI: 1.01, 2.01] [31].

### Stress

Paternal stress exposure was examined in three papers [43, 76, 77]; including one rated as good quality [77]. This study found men's baseline perceived stress scale [PSS] scores were not associated with fecundability [77].

### Nutrition

Papers examining paternal nutrition (*n* = 13) evaluated the associations of a range of nutritional exposures including paternal preconception folate, vitamins B6 and B12, and general dietary patterns with numerous offspring outcomes. These papers utilized data from several studies (*n* = 8) originating in the US, Norway, The Netherlands, and Australia. Approximately half of these papers (*n* = 7) rated as good quality.

Paternal nutritional factors explored included dietary patterns [82, 83] or specific foods groups including dairy [86], and meat [87]. IVF/ICSI-induced live birth was an outcome examined in three papers [82, 86, 87]. A positive association was found between paternal poultry intake and fertilization rate, with a higher fertilization rate among men in the highest quartile of poultry intake [78%] compared with those in the lowest quartile [65%] [87]. Men's total dairy intake was not associated with fertilization rate, implantation rate, clinical pregnancy rate, or live birth rate [86]. Also, paternal adherence to specific dietary patterns [pattern 1 = greater intake of processed foods/meats/high fat/dairy/sugar; pattern 2 = greater intake of fruit/vegetables/legumes/whole grains/nuts/fish] was not associated with fertilization rate [82] when undergoing IVF cycles.

One paper investigated dietary exposures during adolescence and subsequent neonatal health [80]. In a sample of adolescents followed into adulthood becoming fathers (*n* = 2,140), an extra serving of fruit per week was associated with a 2.35 g increase in offspring placenta weight [95% CI: 0.284, 4.42], *P* = 0.03 [80]. Further, paternal lunching regularly in adolescence was associated with an increase in offspring head circumference (β = 0.160, [95% CI: 0.001, 0.320], *P* = 0.05) and whole grain bread consumption was associated with a lower ponderal index (β = -0.003, [95% CI: -0.005, -0.001], *P* = 0.01) [80]. Birthweight was not associated with any paternal dietary exposures [80].

Generally, paternal preconception dietary patterns were collected through paternal self-reports on standardised food frequency questionnaires (FFQ) at baseline and include fast foods [42]; males eating fast food more frequently had infants born earlier than men who eat fast food less frequently (-0.16, [95% CI: − 0.32, 0.00], *P* = 0.04) [42].

Two papers specifically investigated paternal folate [79, 81]. In males undergoing fertility treatment, a 400 μg/day higher preconception folate intake was associated with a 2.6-day longer gestation [95% CI: 0.8, 4.3], *P* = 0.004 [81]. In spontaneously conceived pregnancies, a significant negative association was found between paternal red blood cell [RBC] folate status and crown-rump length (CRL) trajectories, in Quartile 2 [875–1,018 nmol/L;] (β = -0.14; [95% CI:—0.28, -0.006], *P* = 0.04) and Quartile 4 [1,196–4,343 nmol/L] (β =—0.19, [95% CI:—0.33, -0.04], *P* = 0.012) compared with the reference values in Quartile 3 [79]. A negative association was also found for embryonic volume (EV) trajectories in Quartile 4 (β =—0.12, [95% CI: -0.20, -0.05], *P *= 0.001) [79].

## Discussion

This paper reports the first review collating literature assessing modifiable paternal health behaviours and risk factors in the preconception period and highlights clear disparity between the preconception research for women as compared to that for men. While single papers identified in our review do demonstrate adverse pregnancy and offspring outcomes associated with paternal risk factors in the preconception period, current research of paternal health behaviours and risk factors provides an emerging rather than mature evidence-base. Nevertheless, our review did identify a number of important findings.

One consistent finding of this review was the association between paternal preconception smoking and increased risk of adverse infant outcomes, including birth defects and childhood leukemia especially acute myeloid leukemia/acute myeloblastic leukemia (AML). Adverse outcomes such as birth defects are mirrored in maternal preconception smoking literature [89–91], yet the impact of maternal smoking on the risk of AML remains contentious [92, 93]. Smoking in the preconception period may be as perilous for males as for females, as smoking can potentially affect semen quality [94]. Many male smokers (and even more so in smoking couples) consider smoking an indispensable characteristic of their domestic, social and working lives [95] and many report a lack of motivation, willpower, and/or strength to successfully quit [96], in turn influencing female smoking patterns and family environments [95]. Paternal preconception smoking may well be contributing to the estimated 240,000 newborns dying worldwide annually due to birth defects [97]. The finding of paternal preconception smoking and the increased risk of adverse infant outcomes is altogether disconcerting considering the widespread use of tobacco, and that males are more likely than females to engage in risk-taking behaviours, including smoking [98]; the estimated global prevalence of male adolescent smokers in 133 countries is 23.29%.

The papers in this review which focus upon body composition with birthweight outcomes generally affirm positive associations between increasing paternal BMI and offspring birthweight. Indeed, this finding aligns with the literature outside this review which acknowledges that mothers and fathers with overweight or obesity are more likely to have children with overweight or obesity [99–102], compared with those with a normal weight. The positive associations between increasing paternal BMI and offspring bodyweight may, in part, be due to paternal contributions of sperm quality and potential changes to the epigenetic profiles of spermatoza [10, 103] resulting from unhealthy preconception environments and relationships with food. Food-based parenting strategies [100] and spending too much time sedentary [104] may also contribute to influencing offspring weight status. One paper in this review did chart offspring weight and BMI changes from childhood into adulthood [24], however, this reported research did not control for the offspring’s diet and physical exercise.

Nonetheless, an individual’s birthweight can influence both their body weight in childhood [105] and their body weight as they transition into adulthood [106]; external literature positively associates both a higher birthweight and childhood obesity with overweight/obesity at 15–20 years of age [107]. Frameworks to maintain healthy bodyweight, in turn promoting healthy birthweights, endure in the Global action plan on physical activity 2018–2030 [108] and in national overweight/obesity guidelines in countries such as Australia [109] and the Unites States [110].

It is important to note that most papers included in this review utilize retrospective reports (paternal self-reports or maternal reports) of anthropometric data collected at baseline. Such retrospective self-reporting is also evident in the maternal preconception literature [111, 112] and is often considered unreliable and subject to inaccuracies due to self-reporting bias or recall bias [113]. Inaccuracies and reporting bias may be present in particular in papers that utilize maternal reports of paternal preconception height and body weight at minimum 10 weeks of gestation in some papers up to 4 months postpartum. Consequently, retrospective reports of data at baseline may undermine the validity, accuracy, and therefore the reliability of BMI data used in these papers.

The majority of papers in our review report research undertaken in distinct geographical regions with the USA, Europe and the UK, and China heavily represented. As such, the implications for reduced geographical spread of the available research examining paternal preconception health exposures and outcomes must also be considered. It may be that existing region-specific idiosyncrasies of paternal health behaviours, and associated adverse health outcomes for their children, are yet to be described due to the absence of research conducted in other countries and cultures. These gaps limit the opportunities for tailored preconception care policies and interventions and constrain the broader understanding of the potential importance of paternal preconception care. Notwithstanding, such issues foster opportunities for other countries and cultures to identify, learn from and support paternal health.

While almost all papers in this review adjust for some confounders, less than half (*n* = 23) adjusted for the same maternal exposure (i.e., paternal BMI studies adjusting for maternal BMI). Many papers in this review did not adjust for maternal exposures and thus may present biased results and conclusions. Further, many maternal studies do not control for paternal exposures which is a limitation in the field that requires urgent research attention and refocus.

The date parameters set during the search may also represent a limitation as it may have resulted in manuscripts published before the 2012 being overlooked. However, up until recently the preconception research field has primarily focused on the effects of maternal exposures and as such it is unlikely that significant research was overlooked by this date restriction.

Further limitations of the review include the potential for missed citations due to issues with article indexing. Our search protocol did not employ search term truncations or singular synonyms in the final search string which may have resulted in some citations being missed. However, the search protocol was informed by an experienced health librarian, and additional methods – such as reference list and citation checking—were used to identify relevant manuscripts not identified through the primary search. Furthermore, previous search strings trialed for this review that used different synonyms, truncations and search term categories did not result in any additional relevant manuscripts being identified beyond those included in the final search. As such, the literature review is the most comprehensive review of the topic conducted to date.

This review is innovative in that it provides the first examination of paternal preconception risk factors and their association with adverse pregnancy and offspring outcomes. The rigour of the review is also bolstered through adhering to established systematic review reporting guidelines (PRISMA and AMSTAR).

## Conclusion

Overall, this review shows that paternal preconception modifiable risk factors are largely underexplored; smoking and body composition appear to be important areas for consideration in paternal preconception care. While the current literature identifies an emerging evidence-base around paternal preconception modifiable risk factors, there is a need for further investigation to help better inform paternal preconception care and national and international preconception care guidelines. In particular, further research is necessary to identify and better understand the modifiable risk factors affecting males in the preconception period, and how these risk factors influence offspring outcomes, to inform clinical recommendations and health decisions. The future of paternal preconception care and the integration of such care into frontline health practice and policy rests with informed collaboration between clinicians, researchers and policymakers [8].

## Supplementary Information

**Additional file 1.**

## Data Availability

All data extracted for this systematic review are presented as part of the manuscript.

## Abbreviations

AGD: Anogenital distance
BMI: Body mass index
CRL: Crown-rump length
EV: Embryonic volume
FFQ: Food frequency questionnaires
GPs: General practitioners
ICSI: Intracytoplasmic sperm injection
IVF: In-vitro fertilization
LGA: Large for gestational age
NOS: Newcastle–Ottawa Scale [NOS]
POHaD: Paternal Origins of Health and Disease
PICO: Population, Intervention, Comparison, Outcome
PRISMA: Preferred Reporting Items for Systematic Reviews and Meta-Analyses
SGA: Small for gestational age
TTP: Time to pregnancy
TAHS: Tasmanian Longitudinal Health Study
NFPCP: National Free Pre-conception Check-up Projects
RHINESSA: The Respiratory Health in Northern Europe, Spain and Australia multigeneration study
ALL: Childhood acute lymphoblastic leukemia
FEV1: Forced expiratory volume in one second
FVC: Forced vital capacity
CBCL: Child Behaviour Checklist
CBTs: Childhood brain tumours
ECRHS: European Community Respiratory Health Survey
CRCLS: Costa Rican Childhood Leukemia Study
AML: Acute Myeloid Leukemia
FMI: Fat mass index
CL: Childhood acute leukemia
NRCH: National Registry of Childhood Hematopoietic Malignancies
RHINE: Respiratory Heath in Northern Europe
PTB: Preterm birth
AHP: Achieving a Healthy Pregnancy
RRR: Relative risk ratio
OR: Odds ratio
CI: Confidence Interval
PDR: Preconception Dietary Risk Score
HR: Hazard ratio
TL: Telomere length
GZBC: Guangxi Zhaung Birth Cohort
