# Supplementary material for: Paternal preconception modifiable risk factors for adverse pregnancy and offspring outcomes: a review of contemporary evidence from observational studies
Source: BMC Public Health. 2023 Mar 16;23:509. doi: 10.1186/s12889-023-15335-1 (PMC10022288; doi:10.1186/s12889-023-15335-1)
Supplement: Supplementary file 1 — Additional file 1. [file 12889_2023_15335_MOESM1_ESM.docx]

Supplementary File 1 – Search Strategy

1. **Medline (OVID)**

**Controlled vocabulary – Medical subject headings [MESH]**

*af – All fields

*sh – MesH Subject Heading

Ovid MEDLINE(R) and In-Process, In-Data-Review & Other Non-Indexed Citations and Daily <1946 to December 30, 2022>

| **#** | **Query** | **Results from 5^th^ January 2023** |
| --- | --- | --- |
| 1 | (Preconception or pre-conception or periconception or peri-conception or prepregnancy or pre-pregnancy or interconception) .af.  OR  Preconception care .sh.  Limit 1 to yr=”2012-Current” | 11,216 |
| 2 | (Males or fathers or expectant fathers or spouses or husbands or paternal or men’s health) .af.  OR  Male. sh. or men. sh. or fathers. sh. or spouses. sh. or husbands. sh. or Men`s Health. sh.  Limit 1 to yr=”2012-Current” | 3,471,031 |
| 3 | (Infertility or pregnancy outcomes or pregnancy complications or maternal mortality or maternal behavior or maternal health or maternal death or fetal development or fetal death or fetal mortality or fetal weight or perinatal care or perinatal mortality or perinatal death) .af.  OR  Infertility. sh. or infertility, male. sh. or infertility, female. sh. or pregnancy outcome. sh. or pregnancy complications, hematologic. sh. or pregnancy complications, infectious. sh. or pregnancy complications, neoplastic. sh. or pregnancy complications, parasitic. sh. or maternal mortality. sh. or maternal behavior. sh. or maternal health. sh. or maternal death. sh. or fetal development. sh. or fetal death. sh. or fetal mortality. sh. or fetal weight. sh. or perinatal care. sh. or perinatal mortality. sh. or perinatal death.sh.  Limit 1 to yr=”2012-Current” | 153,471 |
| 4 | (Child mortality or child health or child development or infant health or infant death or infant mortality or congenital abnormalities or child behavior disorders or fetal diseases or noncommunicable diseases or life change events) .af.  OR  Child mortality.sh. or child health.sh. or child development.sh. or infant health.sh. or infant death.sh. or infant mortality.sh. or congenital abnormalities.sh. or abnormalities, multiple.sh. or heart defects, congenital.sh. or adverse childhood experiences.sh. or child behavior disorders.sh. or fetal diseases.sh. or noncommunicable diseases.sh. or life change events.sh.  Limit 1 to yr=”2012-Current” | 204,831 |
| 5 | 1 AND 2 AND 3 | 867 |
| 6 | 1 AND 2 AND 4 | 610 |
| 7 | 5 OR 6 | 1,255 |

1. **Embase (OVID)**

**Controlled vocabulary – Science thesaurus [Emtree]**

*af – All fields

*ec – Embase section headings

Embase <1974 to 2022 December 30>

| **#** | **Query** | **Results from 5^th^ January 2023** |
| --- | --- | --- |
| 1 | (Preconception or pre-conception or periconception or peri-conception or prepregnancy or pre-pregnancy or interconception) .af.  OR  Prepregnancy care.ec. or maternal care.ec.  Limit 1 to yr=”2012-Current” | 17,874 |
| 2 | (Males or fathers or expectant fathers or spouses or husbands or paternal or Men`s Health) .af.  OR  Male.ec. or father.ec or expectant father.ec or paternal behavior.ec or paternal exposure.ec or husband.ec.  Limit 1 to yr=”2012-Current” | 440,159 |
| 3 | (Infertility or pregnancy outcomes or pregnancy complications or maternal mortality or maternal behavior or maternal health or maternal death or fetal development or fetal death or fetal mortality or fetal weight or perinatal care or perinatal mortality or perinatal death) .af.  OR  Infertility.ec. or female infertility.ec. or male infertility.ec. or pregnancy outcome.ec. or pregnancy complication.ec. or maternal death.ec. or maternal mortality.ec. or maternal exposure.ec. or fetus development.ec. or perinatal death.ec. or childhood mortality.ec. or childhood disease.ec. or congenital disorder.ec. or congenital malformation.ec. or fetus disease.ec. or fetus death.ec. or fetus mortality.ec.  Limit 1 to yr=”2012-Current” | 176,262 |
| 4 | (Child mortality or child health or child development or infant health or infant death or infant mortality or congenital abnormalities or child behavior disorders or fetal diseases or noncommunicable diseases or life change events) .af.  OR  Infant mortality.ec. or infant disease.ec. or non-communicable disease.ec. or maternal exposure.ec. or adverse outcome.ec. or clinical outcome.ec. or fetus outcome.ec. or patient- reported outcome.ec.  Limit 1 to yr=”2012-Current” | 240,351 |
| 5 | 1 AND 2 AND 3 | 258 |
| 6 | 1 AND 2 AND 4 | 180 |
| 7 | 5 OR 6 | 403 |

1. **Maternity and Infant Care MIDRIS (OVID)**

**Controlled vocabulary – Map Term to Subject Heading**

*af – All fields

*hw – Heading word

Maternity & Infant Care Database (MIDIRS) <1971 to December 13, 2022>

| **#** | **Query** | **Results from 5^th^ January 2023** |
| --- | --- | --- |
| 1 | (Preconception or pre-conception or periconception or peri-conception or prepregnancy or pre-pregnancy or interconception) .af.  OR  Preconception. hw. or pre-conception. hw. or periconception. hw. or peri-conception. hw. or prepregnancy. hw. or pre-pregnancy. hw. or interconception. hw.  Limit 1 to yr=”2012-Current” | 3,959 |
| 2 | (Males or fathers or expectant fathers or spouses or husbands or paternal or Men`s Health) .af.  OR  Males. hw. or fathers. hw or expectant fathers. hw or spouses. hw. or husbands. hw. or paternal. hw or Men`s Health. hw.  Limit 1 to yr=”2012-Current” | 3,842 |
| 3 | (Infertility or pregnancy outcomes or pregnancy complications or maternal mortality or maternal behavior or maternal health or maternal death or fetal development or fetal death or fetal mortality or fetal weight or perinatal care or perinatal mortality or perinatal death or child mortality or child health or child development or infant health or infant death or infant mortality or congenital abnormalities or child behavior disorders or fetal diseases or noncommunicable diseases or or life change events) .af.  Limit 1 to yr=”2012-Current” | 27,070 |
| 4 | Infertility. hw. or pregnancy outcomes. hw. or pregnancy complications. hw. or maternal mortality. hw. or maternal behavior. hw. or maternal health. hw. or maternal death. hw. or fetal development. hw. or fetal death. hw. or fetal mortality. hw. or fetal weight. hw. or or perinatal care. hw. or perinatal mortality. hw. perinatal death. hw. or child mortality. hw. or child health. hw. or child development. hw. or infant health. hw. or infant death. hw. or infant mortality. hw. or congenital abnormalities. hw. or child behavior disorders. hw. or fetal diseases. hw. or noncommunicable diseases. hw. or life change events. hw.  Limit 1 to yr=”2012-Current” | 6,806 |
| 5 | 1 AND 2 AND 3 | 50 |
| 6 | 1 AND 2 AND 4 | 7 |
| 7 | 5 OR 6 | 50 |

1. **CINAHL (EBSCO)**

**Controlled vocabulary – Subject headings [subject headings authority profile]**

*TX – all text

*MW – Word in subject heading

*MM – Major subject heading

Expanders – Apply equivalent subjects

Search modes - Boolean/Phrase

| **#** | **Query** | **Results from 5^th^ January 2023** |
| --- | --- | --- |
| 1 | (TX Preconception or TX pre-conception or TX periconception or TX peri-conception or TX prepregnancy or TX pre-pregnancy or TX interconception)  Published date: 20120101 -20221231 | 6,470 |
| 2 | (MW Preconception or MW pre-conception or MW periconception or MW peri-conception or MW prepregnancy or MW pre-pregnancy or MW interconception)  Published date: 20120101 -20221231 | 1,463 |
| 3 | (MM ”Prepregnancy care”)  Published date: 20120101 -20221231 | 947 |
| 4 | (MM ”Perinatal Care”)  Published date: 20120101 -20221231 | 2,434 |
| 5 | S1 OR S2 OR S3 OR S4 | 8,851 |
| 6 | (TX Males or TX fathers or TX expectant fathers or TX spouses or TX husbands or TX paternal or TX Men`s Health)  Published date: 20120101 -20221231 | 1,189,991 |
| 7 | (MW Males or MW fathers or MW expectant fathers or MW spouses or MW husbands or MW paternal or MW Men`s Health)  Published date: 20120101 -20221231 | 13,166 |
| 8 | (MM ”Expectant Fathers”) or (MM ”Fathers”)  Published date: 20120101 -20221231 | 2,390 |
| 9 | (MM ”Male”)  Published date: 20120101 -20221231 | 103 |
| 10 | or (MM ”Men”) or (MM ”Mens’ Health”)  Published date: 20120101 -20221231 | 7,187 |
| 11 | (MM” Paternal Behavior”) or (MM “Paternal Exposure”)  Published date: 20120101 -20221231 | 259 |
| 12 | (MM “Spouses”)  Published date: 20120101 -20221231 | 4,501 |
| 13 | S6 OR S7 OR S8 OR S9 OR S10 OR S11 OR S12 | 1,191,559 |
| 14 | TX Infertility or TX pregnancy outcomes or TX pregnancy complications or TX maternal mortality or TX maternal behavior or TX maternal health or TX maternal death or TX fetal development or TX fetal death or TX fetal mortality or TX fetal weight or TX perinatal care  Published date: 20120101 -20221231 | 102,235 |
| 15 | TX perinatal mortality or TX perinatal death or  TX child mortality or TX child health or TX child development or TX infant health or TX infant death or TX infant mortality or TX congenital abnormalities or TX child behavior disorders or TX fetal diseases or TX noncommunicable diseases  Published date: 20120101 -20221231 | 84,238 |
| 16 | TX life change events or  MW Infertility or MW pregnancy outcome or MW pregnancy complications or MW maternal mortality or MW maternal behavior or MW maternal health or MW maternal death or  MW fetal development or MW fetal death or MW fetal mortality or MW fetal weight  Published date: 20120101 -20221231 | 49,780 |
| 17 | MW perinatal care or MW perinatal mortality or MW perinatal death or MW child mortality or MW child health or MW child development or MW infant health or MW infant death or MW infant mortality or MW congenital abnormalities or MW child behavior disorders or MW fetal diseases  Published date: 20120101 -20221231 | 53,777 |
| 18 | MW noncommunicable diseases or MW life change events | 5,138 |
| 19 | (MM ”Infertility”)  Published date: 20120101 -20221231 | 7,230 |
| 20 | (MM ”Pregnancy outcomes”) or (MM ”Pregnancy complications, psychiatric”) or (MM ”Pregnancy complications, neoplastic”) or (MM ”Pregnancy complications, hematologic”) or (MM ”Pregnancy complications, parasitic”) or (MM ”Pregnancy complications, infectious”) or (MM ”Pregnancy complications, cardiovascular”)  Published date: 20120101 -20221231 | 13,760 |
| 21 | (MM” maternal behavior”) or (MM ”maternal mortality”) or (MM ”maternal exposure”)  Published date: 20120101 -20221231 | 4,951 |
| 22 | (MM” fetal weight”) or (MM ”fetal abnormalities”) or (MM ”fetal well-being”) or (MM” fetal diseases”) or (MM ”fetal development”)  Published date: 20120101 -20221231 | 4,940 |
| 23 | (MM ”perinatal death”) or (MM ”perinatal care”)  Published date: 201220101 -20221231 | 5,334 |
| 24 | (MM ”child mortality”) or (MM ”child health”) or (MM ”child development disorders”) or (MM ”child development”)  Published date: 201220101 -20221231 | 15,416 |
| 25 | (MM ”infant death”) or (MM ”infant mortality”) or (MM ”infant, premature, diseases”) or (MM ”infant development disorders”)  Published date: 201220101 -20221231 | 4,949 |
| 26 | S14 OR S15 OR S16 OR S17 OR S18 OR S19 OR S20 OR S21 OR S22 OR S23 OR S24 OR S25 | 187,328 |
| 27 | S5 AND S13 AND S26 | 871 |

1. **APA PsycINFO (EBSCO)**

**Controlled vocabulary – APA Thesaurus of Psychological Index Terms**

*TX – all text

*MA – MeSH subject heading

Expanders – Apply related words; Apply equivalent subjects

Search modes – Boolean/Phrase

| **#** | **Query** | **Results from 5^th^ January 2023** |
| --- | --- | --- |
| 1 | TX Preconception or TX pre-conception or TX periconception or TX peri-conception or TX prepregnancy or TX pre-pregnancy or TX interconception or MA prenatal care or MA prenatal exposure  Publication year 2012-2023 | 75,946 |
| 2 | TX Males or TX fathers or TX expectant fathers or TX spouses or TX husbands or TX paternal or TX Men`s Health)  Publication year 2012-2023 | 985,485 |
| 3 | MA human males or MA men or MA fathers or MA expectant fathers or MA husbands or MA spouses  Publication year 2012-2023 | 4,070 |
| 4 | S2 OR S3 | 985,532 |
| 5 | TX Infertility or TX pregnancy outcomes or TX pregnancy complications or TX maternal mortality or TX maternal behavior or TX maternal health or TX maternal death or TX fetal development or TX fetal death or TX fetal mortality or TX fetal weight or TX perinatal care  Publication year 2012-2023 | 21,984 |
| 6 | TX perinatal mortality or TX perinatal death or TX child mortality or TX child health or TX child development or TX infant health or TX infant death or TX infant mortality or TX congenital abnormalities or TX child behavior disorders or TX fetal diseases or TX noncommunicable diseases  Publication year 2012-2023 | 92,934 |
| 7 | TX life change events or MA Infertility or MA pregnancy outcomes or MA mortality risk or MA health outcomes or MA child death or MA congenital disorders or MA death and dying or MA prenatal development or MA infant development or MA child health or MA life span  Publication year 2012-2023 | 15,522 |
| 8 | S5 OR S6 OR S7 | 116,920 |
| 9 | S1 AND S4 AND S8 | 3,031 |

1. **Scopus**

**Controlled vocabulary – *Note Scopus does not use a controlled vocabulary/ subject heading.**

| **#** | **Query** | **Results from 5^th^ January 2023** |
| --- | --- | --- |
| 1 | Preconception or pre-conception or periconception or peri-conception or prepregnancy or pre-pregnancy or interconception AND (LIMIT-TO (PUBYEAR, 2023) OR (LIMIT-TO (PUBYEAR, 2022) OR(LIMIT-TO (PUBYEAR, 2021) OR(LIMIT-TO (PUBYEAR, 2020) OR(LIMIT-TO (PUBYEAR, 2019) OR(LIMIT-TO (PUBYEAR, 2018) OR(LIMIT-TO (PUBYEAR, 2017) OR(LIMIT-TO (PUBYEAR, 2016) OR(LIMIT-TO (PUBYEAR, 2015) OR(LIMIT-TO (PUBYEAR, 2014) OR(LIMIT-TO (PUBYEAR, 2013) OR(LIMIT-TO (PUBYEAR, 2012) | 50,107 |
| 2 | Males or fathers or (expectant AND fathers) or spouses or husbands or paternal or (men’s AND health) AND (LIMIT-TO (PUBYEAR, 2023) OR (LIMIT-TO (PUBYEAR, 2022) OR(LIMIT-TO (PUBYEAR, 2021) OR(LIMIT-TO (PUBYEAR, 2020) OR(LIMIT-TO (PUBYEAR, 2019) OR(LIMIT-TO (PUBYEAR, 2018) OR(LIMIT-TO (PUBYEAR, 2017) OR(LIMIT-TO (PUBYEAR, 2016) OR(LIMIT-TO (PUBYEAR, 2015) OR(LIMIT-TO (PUBYEAR, 2014) OR(LIMIT-TO (PUBYEAR, 2013) OR(LIMIT-TO (PUBYEAR, 2012) | 880,110 |
| 3 | Infertility or pregnancy or (pregnancy AND outcome) or (pregnancy AND complications) or (maternal AND mortality) or (maternal AND behavior) or (maternal AND health) or (maternal AND death) or (fetal AND development) or (fetal AND death) or (fetal AND mortality) or (fetal AND weight) or (perinatal AND care) or (perinatal AND mortality) or (perinatal AND death) or  (child AND mortality) or (child AND health) or (child AND development) or (infant AND health) or (infant AND death) or (infant AND mortality) or (congenital AND abnormalities) or (child AND behavior AND disorders) or (fetal AND diseases) or (noncommunicable AND diseases) or (life AND change AND events) AND  (LIMIT-TO (PUBYEAR, 2023) OR (LIMIT-TO (PUBYEAR, 2022) OR(LIMIT-TO (PUBYEAR, 2021) OR(LIMIT-TO (PUBYEAR, 2020) OR(LIMIT-TO (PUBYEAR, 2019) OR(LIMIT-TO (PUBYEAR, 2018) OR(LIMIT-TO (PUBYEAR, 2017) OR(LIMIT-TO (PUBYEAR, 2016) OR(LIMIT-TO (PUBYEAR, 2015) OR(LIMIT-TO (PUBYEAR, 2014) OR(LIMIT-TO (PUBYEAR, 2013) OR(LIMIT-TO (PUBYEAR, 2012) | 3,643 |
| 4 | 1 AND 2 AND 3 | 383 |

1. **ISI Proceedings**

**Controlled vocabulary – *Note ISI Proceedings [Web of Science] does not use a controlled vocabulary/subject heading.**

***ALL – All fields**

| **#** | **Query** | **Results from 5^th^ January 2023** |
| --- | --- | --- |
| 1 | (ALL = (Preconception) or ALL = (pre-conception) or ALL = (periconception) or ALL = (peri-conception) or ALL = (prepregnancy) or ALL = (pre-pregnancy) or ALL = (interconception)  Publication date 2012-01-01 to 2023-01-05 | 721 |
| 2 | ALL = (males) or ALL = (fathers) or ALL = (expectant fathers) or ALL = (spouses) or ALL = (husbands) or ALL = (paternal) or ALL = (men’s health)  Publication date 2012-01-01 to 2023-01-05 | 26,468 |
| 3 | ALL = (Infertility) or ALL = (pregnancy) or ALL = (pregnancy AND outcome) or ALL = (pregnancy AND complications) or ALL = (maternal AND mortality) or ALL = (maternal AND behavior) or ALL = (maternal AND health) or ALL = (maternal AND death) or ALL = (fetal AND development) or ALL = (fetal AND death) or ALL = (fetal AND mortality) or ALL = (fetal AND weight) or ALL = (perinatal AND care) or ALL = (perinatal AND mortality) or ALL = (perinatal AND death) or  ALL = (child AND mortality) or ALL = (child AND health) or ALL = (child AND development) or ALL = (infant AND health) or ALL = (infant AND death) or ALL = (infant AND mortality) or ALL = (congenital AND abnormalities) or ALL = (child AND behavior AND disorders) or ALL = (fetal AND diseases) or ALL = (noncommunicable AND diseases) or ALL = (life AND change AND events)  Publication date 2012-01-01 to 2023-01-05 | 64,323 |
| 4 | 1 AND 2 AND 3 | 14 |
